# Anomeric 1,2,3-triazole-linked sialic acid derivatives show selective inhibition towards a bacterial neuraminidase over a trypanosome *trans*-sialidase

**DOI:** 10.3762/bjoc.18.24

**Published:** 2022-02-17

**Authors:** Peterson de Andrade, Sanaz Ahmadipour, Robert A Field

**Affiliations:** 1 Manchester Institute of Biotechnology and Department of Chemistry, University of Manchester, 131 Princess Street, Manchester M1 7DN, UK; 2 Iceni Glycoscience Ltd, Norwich Research Park NR4 7GJ, UK

**Keywords:** inhibition, neuraminidase, sialic acid, *trans*-sialidase, 1,2,3-triazole

## Abstract

Sialic acid is the natural substrate for sialidases and its chemical modification has been a useful approach to generate potent and selective inhibitors. Aiming at advancing the discovery of selective *Trypanosoma cruzi trans*-sialidase (TcTS) inhibitors, we have synthesised a small series of anomeric 1,2,3-triazole-linked sialic acid derivatives in good yields and high purity via copper-catalysed azide–alkyne cycloaddition (CuAAC, click chemistry) and evaluated their activity towards TcTS and neuraminidase. Surprisingly, the compounds showed practically no TcTS inhibition, whereas ca. 70% inhibition was observed for neuraminidase in relation to the analogues bearing hydrophobic substituents and ca. 5% for more polar substituents. These results suggest that polarity changes are less tolerated by neuraminidase due to the big difference in impact of hydrophobicity upon inhibition, thus indicating a simple approach to differentiate both enzymes. Moreover, such selectivity might be reasoned based on a possible steric hindrance caused by a bulky hydrophobic loop that sits over the TcTS active site and may prevent the hydrophobic inhibitors from binding. The present study is a step forward in exploiting subtle structural differences in sialidases that need to be addressed in order to achieve selective inhibition.

## Introduction

**Figure 1:**
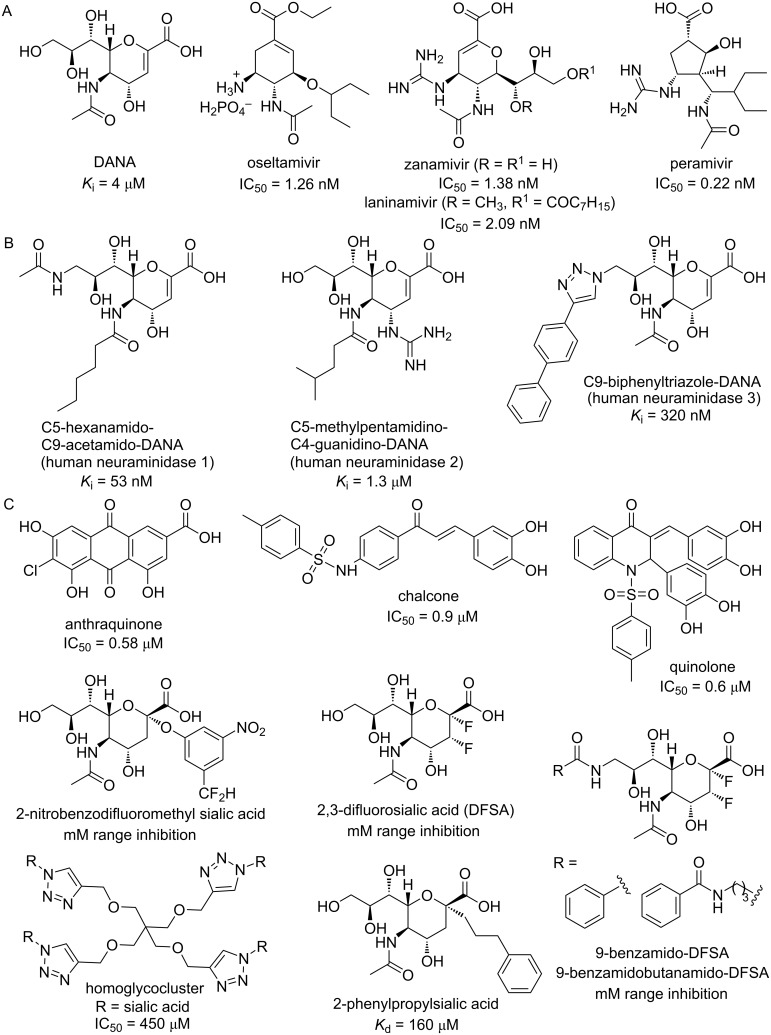
Chemical structures and reported activities of viral (A), human neuraminidases (B) and *Trypanosoma cruzi trans*-sialidase (TcTS) (C) inhibitors.

**Figure 2:**
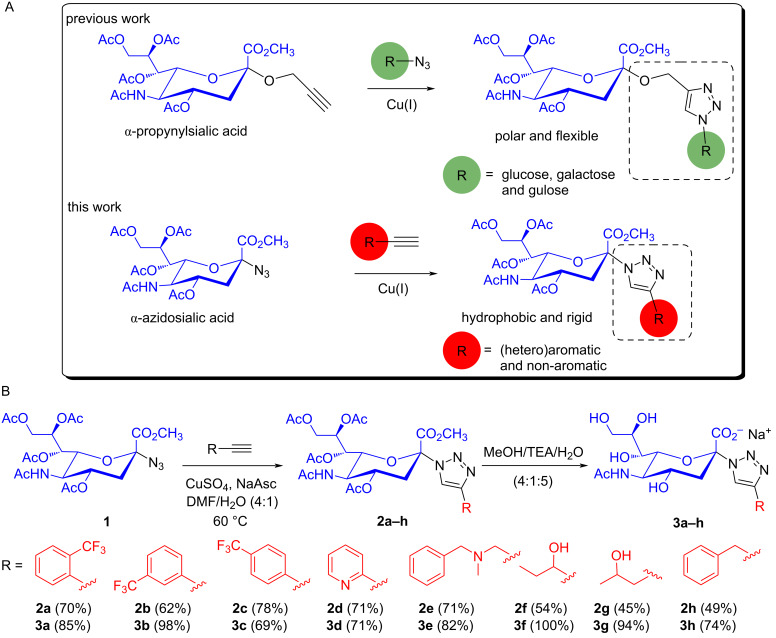
Design and synthesis of potential neuraminidase and *trans*-sialidase inhibitors exploiting a moiety replacement approach considering our previous work (A) and synthesis of 1,2,3-triazole-linked sialic acid derivatives **3a–h** via copper-catalysed azide–alkyne cycloaddition (CuAAC) from the key intermediate **1** (B).

Amongst the diversity of glycans present in living organisms, *N*-acetylneuraminic acid (Neu5Ac, sialic acid) is typically found as a terminal unit of surface glycoconjugates and is crucial to various cellular recognition events in both physiological and pathological processes [1]. This distinctive negatively charged monosaccharide is the natural substrate for sialidases, which belong to different glycoside hydrolase (GH) families and play fundamental roles in the biology of humans, viruses, bacteria and parasitic protozoa by cleaving glycosidic linkages and releasing (or transferring) sialic acid from sialylated substrates [2]. In fact, sialidases have been associated with the pathogenesis of various diseases and the development of potent and selective inhibitors can serve as the basis for new therapeutics [3]. Despite the low primary sequence similarity among human, viral and non-viral sialidases (bacterial and protozoa), they all share a similar catalytic domain with active site residues highly conserved across the species [4–5]. Consequently, the selective inhibition of such important therapeutic targets becomes very difficult. While some progress has been made with respect to viral [6–8] (Figure 1A) and different human neuraminidase isoforms [9–11] (Figure 1B), advances towards bacterial and protozoa sialidases remain a big challenge. An important example relates to *Trypanosoma cruzi trans*-sialidase (TcTS), which plays a central role in the infection process and modulation of the host immune response in Chagas disease. Anchored to the protozoan parasite surface, TcTS transfers terminal sialic acid from the human host glycoconjugates onto its surface mucins to generate α-2,3-linked sialylated β-galactopyranose units, thus contributing directly to the parasite adhesion and invasion of host cells [12]. Although TcTS is the major parasite virulence factor [13], there is no nanomolar inhibitor developed to date. The most potent TcTS inhibitors described are non-carbohydrate-based molecules (anthraquinones [14], chalcones and quinolones [15]) with low micromolar activity, whereas sialic acid-based analogues typically show high millimolar inhibitory activity [16], with few exceptions such as a pentaerythritol homoglycocluster reported by the Carvalho group [17] and a *C*-sialoside bearing phenylpropyl group at C-2 [18] (Figure 1C). In the context of mimicking the terminal sugars α-ᴅ-Neu5Ac(2,3)-β-ᴅ-Gal of *Trypanosoma cruzi* mucins to obtain potent TcTS inhibitors, our group previously synthesised a small series of C-2-modified sialic acid bearing a monosaccharide tethered via 1,2,3-triazole ring (sialylmimetic neoglycoconjugates) [19] that showed 67–91% inhibitory activity at 1 mM. We now envisaged replacing the monosaccharide moiety by (hetero)aromatic substituents (Figure 2A) expecting better inhibition with hydrophobic substituents as observed for the high affinity reported for *C*-sialoside. Additionally, we limited the substituent flexibility by placing the 1,2,3-triazole ring directly at C-2 as a means to mimic the rigidity of the low micromolar non-carbohydrate-based inhibitors. A similar approach has been used to synthesise 1,2,3-triazole-linked sialic acid derivatives at C-2 from various non-aromatic alkynes, among which a long hydrophobic chain showed the best inhibitory activity (IC_50_ = 28 µM) for a bacterial neuraminidase [20]. More recently, a long alkyl chain (benzyl *N*-butylcarbamate) has also been introduced at C-2 of α-triazole-linked sialic acid derivatives modified at C-9 as ligands for the transmembrane glycoprotein CD22 [21]. In this sense, we have synthesised a small series of 1,2,3-triazole-linked sialic acid derivatives via copper-catalysed azide–alkyne cycloaddition (CuAAC, click chemistry), from α-azidosialic acid **1** and commercially available terminal alkynes (Figure 2B), and assessed their inhibitory activity towards TcTS and bacterial neuraminidase.

## Results and Discussion

### Synthesis of sialic acid derivatives

A small series of anomeric 1,2,3-triazole-linked sialic acid derivatives was synthesised as outlined in Figure 2B. Emulating our previous work with anomeric azide CuAAC click chemistry [17,22–24], the well-known α-azidosialic acid **1** [25] was synthesised from *N*-acetylneuraminic acid in four steps [26] in good overall yield (55%). The assignment of the anomeric configuration of **1** was based on the chemical shift of H_3eq_, which is located in lower magnetic field (over 2.5 ppm) when compared to a β-glycoside (under 2.5 ppm) [27]. Additionally, the anomeric configuration can be determined through the coupling pattern of C-1 in a selective proton decoupled ^13^C NMR experiment, where the α-anomer is a doublet and the β-anomer is a singlet [28]. The key intermediate **1** was further used in CuAAC reaction [29–32] with eleven (hetero)aromatic and non-aromatic terminal alkynes readily available in our lab [23].

Although CuAAC is reputedly tolerant of a broad range of substrates, solvents, and reaction conditions; all these parameters have to be carefully planned to avoid low yields or even no product formation, as previously described for compound **1** [20]. Amongst the vast number of reported procedures, the 1,3-dipolar cycloaddition was performed with 20 mol % excess of the terminal alkynes in a mixture of solvents (DMF/H_2_O 4:1) at 60 °C and Cu(I) generated in situ [33], but in catalytic amount. This approach resulted in the synthesis of eight 1,4-disubstituted 1,2,3-triazole derivatives (**2a**–**h**) in good yields (45–78%) and high purity. Other two reactions proceeded to completion but the starting material **1** and the products have very similar *R*_f_ values, making purification difficult. Consequently, these compounds were obtained only as mixture with starting material due to purification issues and were not considered for further evaluation. The structure of compounds **2a**–**h** were confirmed by ^1^H and ^13^C NMR spectroscopy (see spectra in Supporting Information File 1) as well as HRESIMS analyses. The triazole ring hydrogen was observed as a singlet in the range δ_H_ 7.7–8.5 ppm and its corresponding carbon (CH-triazole) in the range δ_C_ 120–123 ppm, consistent with the spectra of 1,4-disubstituted triazole regiochemistry [22]. The final step was carried out in CH_3_OH/triethylamine/H_2_O 4:1:5 [26], followed by triethylammonium ion exchange for Na^+^ upon treatment with Amberlite IR 120 (Na^+^), to give the fully deprotected derivatives **3a–h** in excellent yields and purity (qualitative assessment based on the NMR analysis) without further purification.

### Enzyme inhibition assays

**Figure 3:**
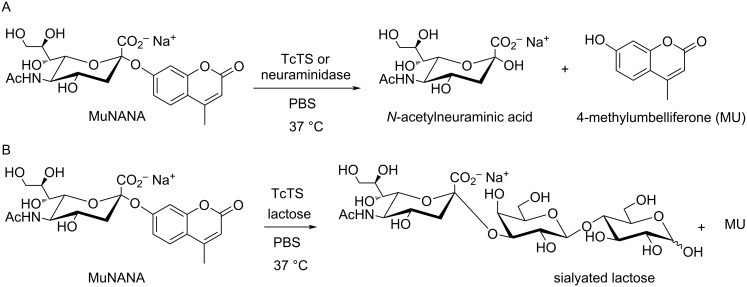
TcTS and neuraminidase hydrolase activity (A) as well as TcTS transferase activity (B) in the presence of an acceptor substrate.

The inhibitory activities of compounds **3a–h** toward TcTS and neuraminidase were assessed by a continuous fluorimetric assay [34], which is based on the residual hydrolase activity of both enzymes (Figure 3A) (and TcTS transferase activity in the presence of an acceptor substrate, such as lactose – Figure 3B) by releasing the fluorophore 4-methylumbelliferone (MU) for detection upon cleavage of the substrate 2'-(4-methylumbelliferyl) α-ᴅ-*N*-acetylneuraminic acid (MUNANA).

**Figure 4:**
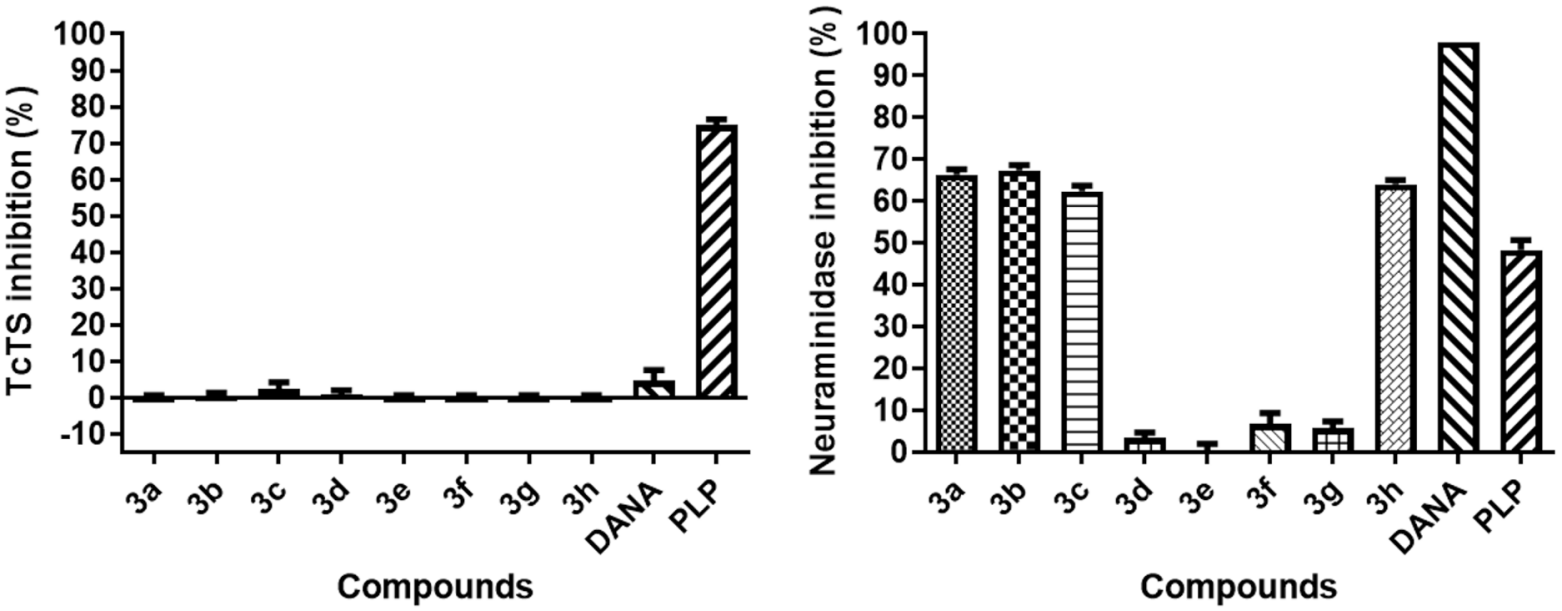
TcTS and neuraminidase inhibition by 1,2,3-triazole-linked sialic acid derivatives **3a–h** (1 mM) using a fluorimetric assay. DANA (2,3-dehydro-2-deoxy-*N*-acetylneuraminic acid) and PLP (pyridoxal phosphate) were used as positive controls at 1 mM.

Compounds **3a**–**h** were tested at 1.0 mM in the presence of the donor substrate MUNANA (0.1 mM) along with pyridoxal phosphate (PLP, *K*_i_ = 7.3 mM) [35] and 2,3-dehydro-2-deoxy-*N*-acetylneuraminic acid (DANA) [36] as positive controls for TcTS and neuraminidase, respectively. Surprisingly, the results showed practically no inhibitory activity for TcTS, whereas ca. 70% inhibition was observed for neuraminidase in relation to compounds **3a**–**c** and **3h** (Figure 4). Although the small number of compounds tested does not allow a comprehensive structure–activity relationship analysis, it is interesting to notice that hydrophobicity is important to inhibition of neuraminidase as the most potent compounds possess hydrophobic aromatic substituents. Conversely, increase in polarity results in weak inhibition (ca. 5%) as noted for compounds bearing substituents with nitrogen or a hydroxy group (**3d**–**g**). In fact, the higher polarity of compounds **3d**–**g** is expressed by their lower LogP (−0.84 to −2.67) compared to the more hydrophobic compounds **3a**–**c** and **3h** (−0.56 to 0.08) (see Table S1 in Supporting Information File 1).

**Figure 5:**
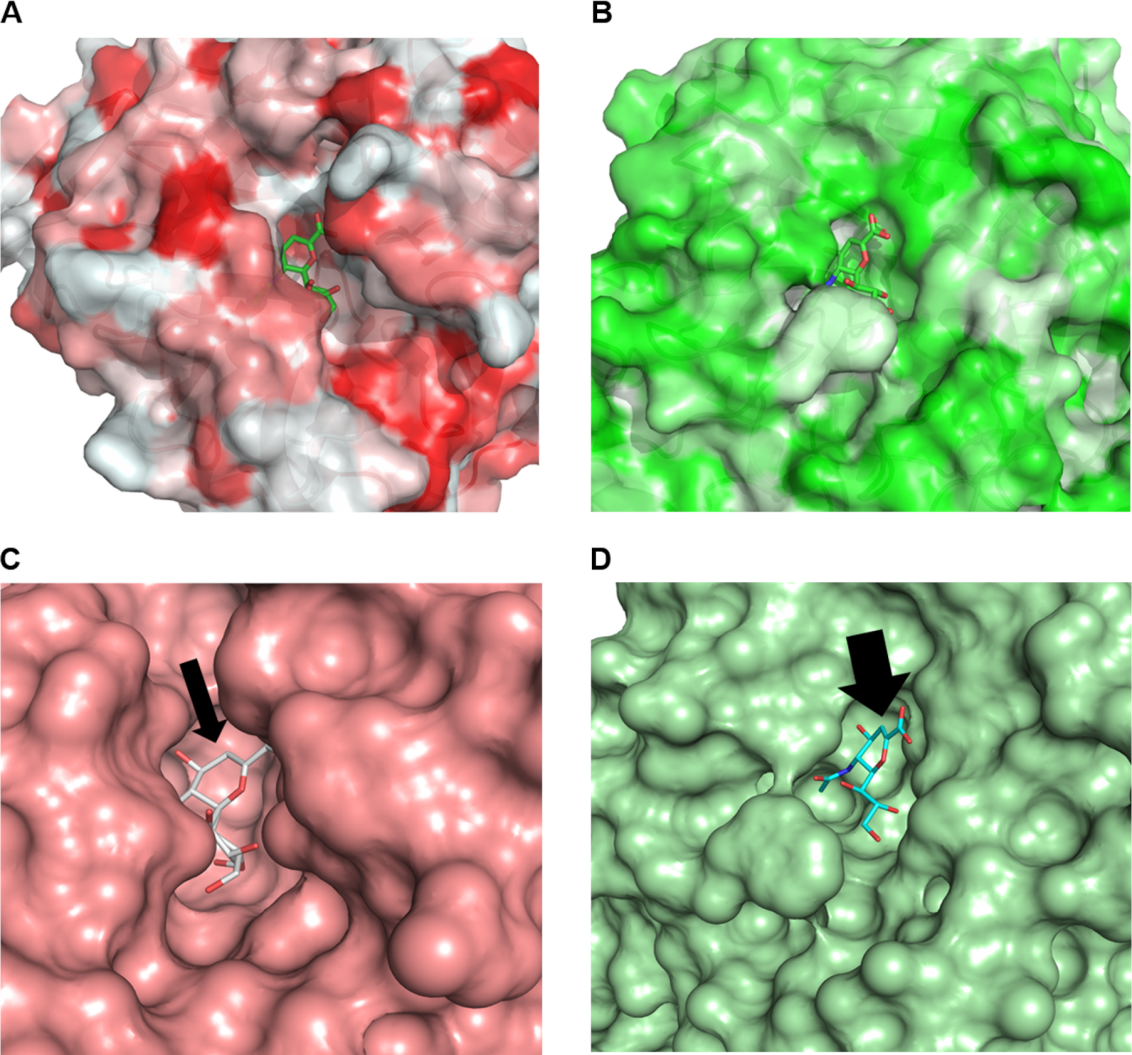
Crystal structure of TcTS (PDB code 1MS1 – coloured red) (A) and neuraminidase (PDB code 2VK6 – coloured green) (B) in complex with DANA showing a bulky hydrophobic loop that sits over TcTS active site in its absence for neuraminidase. The black arrows indicate the difference between the narrow (TcTS) (C) and wide (neuraminidase) (D) regions around C-2 in both active sites, possibly preventing the inhibitors from entering the TcTS active site due to steric hindrance.

Given the big difference in impact of hydrophobic groups upon inhibition, these results suggest a structural consensus that may lead to a simple approach to differentiate TcTS and neuraminidase inhibitory activity as a polarity change seems to be less tolerated by the latter. This intriguing result is difficult to explain since all sialidases share a very similar catalytic domain, despite low sequence similarity [4]. For instance, hydrophobic pockets in the glycerol- and acetamide-binding subsites have been reported for neuraminidases [10,37] as well as for TcTS, which has a more spacious and hydrophobic active site around C9 of sialic acid [16]. Nonetheless, a simple comparison from the crystal structures of both enzymes in complex with DANA reveals a bulky hydrophobic loop that sits over the active site of TcTS (PDB code 1MS1 – coloured red) (Figure 5A) but is absent for neuraminidase (PDB code 2VK6 – coloured green) (Figure 5B). In this case, induced structural rearrangements caused DANA to be buried in a deep and narrow cavity. Such conformational change could potentially prevent the hydrophobic inhibitors from entering the TcTS active site due to steric hindrance. In this context, our results suggest that the key interactions with the hydrophobic substituents at C-2 have occurred in the less sterically hindered active site (comparison shown with black arrows – Figure 5C and Figure 5D), which in turn conferred selectivity towards the neuraminidase. From the analogues perspective, the absence of TcTS inhibition could be also attributed to the lack of flexibility of the substituents rather than their polarity. Such rigidity posed by the 1,2,3-triazole ring directly bound at C-2 might compromise the most favourable orientations toward crucial interactions of the substituents in the active site. Therefore, this approach was not as promising as expected for TcTS. Regarding the positive control, it is known that PLP is a TcTS weak inhibitor [35,38] and its inhibition does not involve formation of a Schiff-base intermediate [38]. However, an allosteric modulation of neuraminidase activity has been attributed to a selective modification of murine respirovirus neuraminidase via specific PLP-Lysine binding [39]. Although PLP is not a reported neuraminidase inhibitor, its main interaction in the active site could be reasoned based on previous results with sialic acid-derived phosphonate analogues. In this regard, it has been suggested that the inhibition of different strains of influenza virus neuraminidase is due to a strong electrostatic interaction between the phosphonate group and the arginine pocket in the active site [40].

## Conclusion

A small series of anomeric 1,2,3-triazole-linked sialic acid derivatives was synthesised in good yields and high purity via CuAAC click chemistry and evaluated for their potential inhibitory activity towards TcTS and neuraminidase. Unexpectedly, none of the sialic acid derivatives inhibited TcTS. Conversely, the derivatives bearing hydrophobic substituents showed ca. 70% inhibition for neuraminidase, whereas more polar substituents gave rise to weak inhibition (ca. 5%). These results suggest a simple approach to differentiate TcTS and neuraminidase as polarity changes are less tolerated by the latter. Furthermore, the selectivity conferred to neuraminidase might be related to conformational changes of a bulky hydrophobic loop that sits over TcTS active site causing steric hindrance, probably preventing the hydrophobic inhibitors from entering its active site. Also, the absence of TcTS inhibition could potentially be attributed to the lack of flexibility of the substituents, thus compromising key favourable orientations for strong binding in the active site. Despite being a preliminary outcome, the present study has advanced one more step in exploiting the sialidases subtle structural differences to tackle selective inhibition.

## Experimental

### General materials and methods

Chemicals were commercially acquired as reagent grade and used without further purification. *N*-Acetylneuraminic acid was purchased from Carbosynth (MA00746), terminal alkynes were purchased from Sigma-Aldrich, MUNANA [2'-(4-methylumbelliferyl)-α-ᴅ-*N*-acetylneuraminic acid sodium salt hydrate] (BIB6114) was purchased from Apollo Scientific Ltd, pyridoxal 5′-phosphate hydrate (P3657) and DANA (D9050) were purchase from Sigma-Aldrich. Neuraminidase from *Clostridium perfringens* (*C. Welchii*) was purchased from Sigma-Aldrich (N2876-6U) and Milli-Q water was used to prepare all buffers. Thin-layer chromatography (TLC) was performed on pre-coated silica gel 60 F_254_ plates (Merck) and compounds were visualised by UV irradiation (λ = 254 nm) and/or dipping in ethanol/sulfuric acid (95:5 v/v) followed by heating. A Biotage SP4 flash chromatography system was used for purification of the protected sugars with normal phase silica (pre-packed SNAP Ultra cartridges). Deprotected sugars (final products) were lyophilised using a Büchi Lyovapor L-200 freeze dryer. ^1^H, COSY, ^13^C, DEPT-135 and HSQC NMR spectra were recorded on a Bruker Avance III 400 MHz spectrometer at 298 K. Chemical shifts (δ) recorded in CDCl_3_ and D_2_O are reported with respect to the solvent residual peak at 7.26 and 4.79 ppm in ^1^H NMR, respectively. High-resolution mass spectra were acquired using electrospray ionisation in a Waters Vion spectrometer with Waters Acquity LC–MS (positive mode). Fluorescence measurements were performed on a FLUOstar Omega Multi-Mode Microplate Reader.

### Expression and purification of *Trypanosoma cruzi trans*-sialidase (TcTS)

A recombinant *T. cruzi trans*-sialidase (TcTS) plasmid containing the pTrcHisA TcTS 6 11/2 expression construct [41] was transformed into *E. coli* BL21 (DE3) cells and inoculated in 1 L of LB medium containing the transformant and ampicillin (100 μg/mL). Incubation at 37 °C along with shaking (200 rpm) was continued until optical density (OD_600_) reached 0.6. Heterologous protein expression was induced by adding isopropyl β-ᴅ-1-thiogalactopyranoside (IPTG) to a final concentration of 1 mM and incubating for 4 hours at 30 °C with shaking (180 rpm). The cells were harvested by centrifugation (4,000*g*, 20 min), re-suspended in lysis buffer (20 Mm Tris/HCl, pH 8.0, EDTA-free protease inhibitor cocktail tablet, 0.02 mg/mL DNaseI). Cell lysis was performed by sonication on ice. Cells were exposed to Amplitude microns of ultrasound every 20 seconds for 10 minutes. The recombinant protein was separated from cell debris by centrifugation (20,000*g*, 30 min). The supernatant was loaded to a 5 mL HisTrap^TM^ HP column (GE healthcare) pre-equilibrated with buffer A (50 mM Tris-HCl, pH 8.0, 150 mM NaCl, 20 mM imidazole) and purified at 4 °C using an ÄKTA pure FPLC system (GE Healthcare). The column was washed with buffer A to remove unbound proteins followed by elution of bound proteins with buffer B (50 mM Tris-HCl, pH 8.0, 150 mM NaCl, 500 mM imidazole). Further purification was carried out by gel filtration chromatography (Superdex S200 16/600 column, GE Healthcare) with 20 mM HEPES, pH 7.5, 150 mM NaCl, 1 mL/min. Fractions containing TcTS were pooled and concentrated using Amicon Ultra-15 Centrifugal Filter (30,000 MW cut off) [42].

### TcTS and neuraminidase inhibition assays

Inhibition of both enzymes was assessed based on the continuous fluorimetric assay described by Neres and co-workers [34]. Briefly, TcTS assay was performed in duplicate in 96-well plates containing 200 mM phosphate buffer solution pH 7 (20 μL), 0.8 mg/mL recombinant enzyme (20 μL), 5 mM lactose (20 μL) and 5 mM inhibitor (20 μL) solutions. This mixture was kept for 10 min at 25 °C, followed by addition of 0.5 mM MUNANA (20 μL, 0.1 mM final concentration) and incubated at 37 °C for 15 min. The fluorescent product released (MU) was measured with excitation and emission wavelengths of 360 and 460 nm, respectively. Neuraminidase (10 mU) assay was performed as described above without lactose and with 40 μL buffer. The data from three independent experiments were analysed with GraphPad Prism software version 4.0 (San Diego, CA, USA). Inhibition percentages were calculated according to the equation: % *I* = 100 [1 (*V*_i_/*V*_0_)], where *V*_i_ is the velocity in the presence of inhibitor and *V*_0_ is the velocity in absence of inhibitor.

### General procedure to obtain the corresponding 1,2,3-triazole-linked sialic acid derivatives

Sodium ascorbate (6 mg, 30 µmol) and CuSO_4_ (1 mg, 6 µmol) (6 μL of 1.0 M aq solution) were added to a solution of compound **1** [25] (31 mg, 60 µmol) – synthesised in four steps from *N*-acetylneuraminic acid [26] – and terminal alkyne (72 µmol) in DMF/H_2_O 4:1 (1 mL) in a glass vial. The mixture was stirred for 24 h at 60 °C [33] and solvents were evaporated under vacuum with addition of toluene (3 × 5 mL). EtOAc (10 mL) was added to the crude and washed with H_2_O (3 × 5 mL). The organic layer was dried over MgSO_4_, filtered, concentrated under vacuum and purified by flash chromatography [cartridge: SNAP Ultra 10g; isocratic: 80–80% and 100–100% EtOAc/hexane (v:v); flow: 12 mL/min] to afford 1,4-disubstituted 1,2,3-triazole derivatives **2a**–**h** in good yields and purity. After the deprotection step with CH_3_OH/triethylamine/H_2_O 4:1:5 [26], triethylammonium ions were exchanged upon treatment with Amberlite IR 120 (Na^+^ form) and compounds **3a**–**h** were obtained in excellent yield and purity without further purification.

## Supporting Information

File 1 Analytical data, ^1^H and ^13^C NMR spectra of compounds **1**, **2a–h** and **3a–h**, and calculated LogP of compounds **3a–h**.
