# Supplementary material for: Anomeric 1,2,3-triazole-linked sialic acid derivatives show selective inhibition towards a bacterial neuraminidase over a trypanosome trans-sialidase
Source: Beilstein J Org Chem. 2022 Feb 17;18:208–16. doi: 10.3762/bjoc.18.24 (PMC8895027; doi:10.3762/bjoc.18.24)
Supplement: File 1 — Analytical data, 1H and 13C NMR spectra of compounds 1, 2a–h and 3a–h, and calculated LogP of compounds 3a–h. [file Beilstein_J_Org_Chem-18-208-s001.pdf]

BEILSTEIN JOURNAL OF ORGANIC CHEMISTRY

Supporting Information

for

Anomeric 1,2,3-triazole-linked sialic acid derivatives show selective inhibition towards a bacterial neuraminidase over a trypanosome *trans*-sialidase

Peterson de Andrade, Sanaz Ahmadipour and Robert A. Field

Beilstein J. Org. Chem. **2022,** *18,* 208–216. doi:10.3762/bjoc.18.24

Analytical data, ^{1}H and ^{13}C NMR spectra of compounds 1, 2a–h and 3a–h, and calculated LogP of compounds 3a–h

License and Terms: This is a supporting information file under the terms of the Creative Commons Attribution License (https://creativecommons.org/licenses/by/4.0). Please note that the reuse, redistribution and reproduction in particular requires that the author(s) and source are credited and that individual graphics may be subject to special legal provisions.
The license is subject to the *Beilstein Journal of Organic Chemistry* terms and conditions: (https://www.beilstein-journals.org/bjoc/terms)

CONTENT

Analytical data of compounds **1**, **2a–h** and **3a–h**	S2
^{1}H NMR and ^{13}C NMR spectra of compound **1**	S9
^{1}H NMR and ^{13}C NMR spectra of compound **2a**	S10
^{1}H NMR and ^{13}C NMR spectra of compound **2b**	S11
^{1}H NMR and ^{13}C NMR spectra of compound **2c**	S12
^{1}H NMR and ^{13}C NMR spectra of compound **2d**	S13
^{1}H NMR and ^{13}C NMR spectra of compound **2e**	S14
^{1}H NMR and ^{13}C NMR spectra of compound **2f**	S15
^{1}H NMR and ^{13}C NMR spectra of compound **2g**	S16
^{1}H NMR and ^{13}C NMR spectra of compound **2h**	S17
^{1}H NMR and ^{13}C NMR spectra of compound **3a**	S18
^{1}H NMR and ^{13}C NMR spectra of compound **3b**	S19
^{1}H NMR and ^{13}C NMR spectra of compound **3c**	S20
^{1}H NMR and ^{13}C NMR spectra of compound **3d**	S21
^{1}H NMR and ^{13}C NMR spectra of compound **3e**	S22
^{1}H NMR and ^{13}C NMR spectra of compound **3f**	S23
^{1}H NMR and ^{13}C NMR spectra of compound **3g**	S24
^{1}H NMR and ^{13}C NMR spectra of compound **3h**	S25
Table S1 - Calculated LogP of compounds **3a–h**	S26

Analytical data of compounds 1, 2a–h and 3a–h

Compound **1**:

Methyl 4,7,8,9-tetra-*O*-acetyl-2-azido-5-acetamido-2,3,5-trideoxy-D-*glycero*-α-D-galactonon-2-ulopyranosonate (**1**) [25,26]

Yield: 72% (370 mg; 0.72 mmol). ^{1}H NMR (400 MHz, $CDCl_3$) δ 5.36-5.31 (2H, m, H-8, H-7), 5.29 (1H, d, $J_{\mathrm{AcN}H,5}$ = 10.0 Hz, CH_3CONH), 5.05 (1H, ddd, $J_{4,3ax}$ = 11.7 Hz, $J_{4,5}$ = 10.3 Hz, $J_{4,3eq}$ = 4.8 Hz, H-4), 4.38-4.32 (1H, m, H-9a), 4.16-4.10 (1H, m, H-9b), 4.05 (1H, q, $J_{5,4}$ = $J_{5,\mathrm{AcN}H}$ = $J_{5,6}$ = 10.3 Hz, H-5), 3.91-3.86 (4H, m, H-6, CO_2CH_3), 2.56 (1H, dd, $J_{3eq,3ax}$ = 13.1 Hz, $J_{3eq,4}$ = 4.8 Hz, H-3_{eq}), 2.14 (3H, s, CH_3CO), 2.12 (3H, s, CH_3CO), 2.03 (6H, s, 2x CH_3CO), 1.88 (3H, s, CH_3CONH), 1.87-1.79 (1H, m, H-3_{ax}). ^{13}C NMR (101 MHz, $CDCl_3$) δ 170.9, 170.7, 170.4, 170.2, 170.1 (4x $CH_3\underline{C}O$, C-1), 167.2 ($CH_3\underline{C}ONH$), 89.1 (C-2), 74.1 (C-6), 69.6 (C-8), 68.9 (C-4), 67.6 (C-7), 62.2 (C-9), 53.6 ($CO_2\underline{C}H_3$), 49.4 (C-5), 36.7 (C-3), 23.3 ($\underline{C}H_3CONH$), 21.1, 20.9, 20.8, 20.8 (4x $\underline{C}H_3CO$). HRMS (ESI): *m/z* calculated for $C_{20}H_{28}N_4NaO_{12}$ $[M+Na]^+$: 539.1601; found: 539.1608.

Compounds **2a–h**:

Methyl 4,7,8,9-tetra-*O*-acetyl-2-{4-[2-(trifluoromethyl)phenyl]-1*H*-1,2,3-triazol-1-yl}-5-acetamido-2,3,5-trideoxy-D-*glycero*-α-D-*galacto*non-2-ulopyranosonate (**2a**)

Yield: 70% (28 mg; 0.041 mmol). ^{1}H NMR (400 MHz, $CDCl_3$) δ 8.15 (1H, s, C*H*-triazole), 7.88 (1H, d, *J* = 7.7 Hz, H-Ph), 7.76 (1H, dd, *J* = 8.0 Hz, *J* =1.3 Hz, H-Ph), 7.63 (1H, td, *J* = 7.7, 1.3 Hz, H-Ph), 7.50 (1H, t, *J* = 7.7 Hz, H-Ph), 5.47-5.37 (3H, m, H-8, H-7, CH_3CONH), 5.23 (1H, ddd, *J* = 11.8 Hz, *J* = 10.2 Hz, *J* = 4.5 Hz, H-4), 4.41 (1H, dd, *J* = 10.8 Hz, *J* = 2.2 Hz, H-6), 4.25 (1H, dd, *J* = 12.4 Hz, *J* = 2.6 Hz, H-9a), 4.15 (1H, q, *J* = 10.3 Hz, H-5), 4.08 (1H, dd, *J* = 12.5 Hz, *J* = 5.6 Hz, H-9b), 3.79 (3H, s, CO_2CH_3), 3.48 (1H, dd, *J* = 13.3 Hz, *J* = 4.5 Hz, H-3_{eq}), 2.80 (1H, dd, *J* = 13.4 Hz, *J* = 11.8 Hz, H-3_{ax}), 2.15 (3H, s, CH_3CO), 2.13 (3H, s, CH_3CO), 2.08 (3H, s, CH_3CO), 2.03 (3H, s, CH_3CO), 1.92 (3H, s, CH_3CONH). ^{13}C NMR (101 MHz, $CDCl_3$) δ 171.0, 170.7, 170.4, 170.2, 170.2 (4x $CH_3\underline{C}O$, C-1), 166.7 ($CH_3\underline{C}ONH$), 145.2 ($\underline{C}$CH-triazole), 132.1 (Ph-$\underline{C}$H), 132.0 (Ph-$\underline{C}$H), 129.2 ($C_{quat.}$), 128.7 (Ph-$\underline{C}$H), 128.3 ($C_{quat.}$), 127.9 ($C_{quat.}$), 126.4 (Ph-$\underline{C}$H), 122.3 ($\underline{C}$H-triazole), 88.6 (C-2), 74.0 (C-6), 68.7 (C-4), 68.3 (C-8), 67.1 (C-7), 62.4 (C-9), 54.1 ($CO_2\underline{C}H_3$), 49.5 (C-5), 36.0 (C-3), 23.3 ($\underline{C}H_3CONH$), 21.2, 21.0, 20.9, 20.8 (4x $\underline{C}H_3CO$). HRMS (ESI): *m/z* calculated for $C_{29}H_{33}F_3N_4NaO_{12}$ $[M+Na]^+$: 709.1945; found: 709.1925.

Methyl 4,7,8,9-tetra-*O*-acetyl-2-{4-[3-(trifluoromethyl)phenyl]-1*H*-1,2,3-triazol-1-yl}-5-acetamido-2,3,5-trideoxy-D-*glycero*-α-D-*galacto*non-2-ulopyranosonate (**2b**)

Yield: 62% (28 mg; 0.041 mmol). ^{1}H NMR (400 MHz, $CDCl_3$) δ 8.43 (1H, s, C*H*-triazole), 8.21 (1H, d, *J* = 1.8 Hz, H-Ph), 8.09 (1H, dt, *J* = 7.2 Hz, *J* =1.7 Hz, H-Ph), 7.62-7.53 (2H, m, H-Ph), 5.50 (1H, ddd, *J* = 8.0 Hz, *J* = 6.4 Hz, *J* = 2.7 Hz, H-8), 5.44-5.38 (2H, m, CH_3CONH, H-7), 5.20 (1H, ddd, *J* = 12.0 Hz, *J* = 10.3 Hz, *J* = 4.4 Hz, H-4), 4.44-4.34 (2H, m, H-6, H-9a), 4.16 (1H, q, *J* = 10.4 Hz, H-5), 4.03 (1H, dd, *J* = 12.4 Hz, *J* = 6.4 Hz, H-9b), 3.80 (3H, s, CO_2CH_3), 3.54 (1H, dd, *J* = 13.2 Hz, *J* = 4.4 Hz, H-3_{eq}), 2.73 (1H, dd, *J* = 13.3 Hz, *J* = 12.0 Hz, H-3_{ax}), 2.19 (3H, s, CH_3CO), 2.12 (3H, s, CH_3CO), 2.09 (3H, s, CH_3CO), 2.08 (3H, s, CH_3CO), 1.91 (3H, s, CH_3CONH). ^{13}C NMR (101 MHz, $CDCl_3$) δ 171.0, 170.9, 170.7, 170.4, 170.2 (4x $CH_3\underline{C}O$, C-1), 166.6 ($CH_3\underline{C}ONH$), 147.2 ($\underline{C}$CH-triazole), 131.2 ($C_{quat.}$), 131.1 ($C_{quat.}$), 129.5 (Ph-$\underline{C}$H), 129.1 (Ph-$\underline{C}$H), 125.4 ($C_{quat.}$), 125.1 (Ph-$\underline{C}$H), 122.9 (Ph-$\underline{C}$H), 119.8 (triazole-$\underline{C}$H), 88.7 (C-2), 74.3 (C-6), 68.7 (C-4), 68.6 (C-8), 67.4 (C-7), 62.5 (C-9), 54.3 ($CO_2\underline{C}H_3$), 49.4 (C-5), 36.0 (C-3), 23.3 ($\underline{C}H_3CONH$), 21.4, 21.0, 20.9, 20.8 (4x $\underline{C}H_3CO$). HRMS (ESI): *m/z* calculated for $C_{29}H_{33}F_3N_4NaO_{12}$ $[M+Na]^+$: 709.1945; found: 709.1923.

Methyl 4,7,8,9-tetra-*O*-acetyl-2-{4-[4-(trifluoromethyl)phenyl]-1*H*-1,2,3-triazol-1-yl}-5-acetamido-2,3,5-trideoxy-D-*glycero*-α-D-*galacto*non-2-ulopyranosonate (**2c**)

Yield: 78% (24 mg; 0.035 mmol). ^{1}H NMR (400 MHz, $CDCl_3$) δ 8.38 (1H, s, C*H*-triazole), 8.03 (2H, d, *J* = 8.1 Hz, H-Ph), 7.69 (2H, d, *J* = 8.2 Hz, H-Ph), 5.51 (1H, ddd, *J* = 8.6 Hz, *J* = 5.9 Hz, *J* = 2.7 Hz, H-8), 5.43-5.35 (2H, m, CH_3CONH, H-7), 5.20 (1H, ddd, *J* = 12.0 Hz, *J* = 10.3 Hz, *J* = 4.4 Hz, H-4), 4.40-4.31 (2H, m, H-6, H-9a), 4.15 (1H, q, *J* = 10.3 Hz, H-5), 4.06 (1H, dd, *J* = 12.4 Hz, *J* = 5.9 Hz, H-9b), 3.79 (3H, s, CO_2CH_3), 3.54 (1H, dd, *J* = 13.2 Hz, *J* = 4.5 Hz, H-3_{eq}), 2.72 (1H, dd, *J* = 13.2 Hz, *J* = 12.0 Hz, H-3_{ax}), 2.20 (3H, s, CH_3CO), 2.11 (3H, s, CH_3CO), 2.09 (3H, s, CH_3CO), 2.08 (3H, s, CH_3CO), 1.92 (3H, s, CH_3CONH). ^{13}C NMR (101 MHz, $CDCl_3$) δ 171.0, 170.8, 170.7, 170.4, 170.2 (4x $CH_3\underline{C}O$, C-1), 166.6 ($CH_3\underline{C}ONH$), 147.2 ($\underline{C}$CH-triazole), 133.6 ($C_{quat.}$), 130.5 ($C_{quat.}$), 130.2 ($C_{quat.}$), 126.2 (2x Ph-$\underline{C}$H), 126.0 (2x Ph-$\underline{C}$H), 120.0 (triazole-$\underline{C}$H), 88.6 (C-2), 74.1 (C-6), 68.5 (C-4), 68.2 (C-8), 67.1 (C-7), 62.5 (C-9), 54.3 ($CO_2\underline{C}H_3$), 49.4 (C-5), 36.0 (C-3), 23.3 ($\underline{C}H_3CONH$), 21.4, 21.0, 20.9, 20.9 (4x $\underline{C}H_3CO$). HRMS (ESI): *m/z* calculated for $C_{29}H_{33}F_3N_4NaO_{12}$ $[M+Na]^+$: 709.1945; found: 709.1921.

Methyl 4,7,8,9-tetra-*O*-acetyl-2-[4-(pyridine-2-yl)-1*H*-1,2,3-triazol-1-yl]-5-acetamido-2,3,5-trideoxy-D-*glycero*-α-D-*galacto*non-2-ulopyranosonate (**2d**)

Yield: 71% (30 mg; 0.048 mmol). ^{1}H NMR (400 MHz, $CDCl_3$) δ 8.62 (1H, dt, *J* = 4.9 Hz, *J* = 1.3 Hz, H-Py), 8.50 (1H, s, C*H*-triazole), 8.13 (1H, dd, *J* = 7.9 Hz, *J* = 1.2 Hz, H-Py), 7.77 (1H, td, *J* = 7.7 Hz, *J* = 1.8 Hz, H-Py), 7.25-7.20 (1H, m, H-Py), 5.50 (1H,

ddd, *J* = 8.4 Hz, *J* = 5.3 Hz, *J* = 2.9 Hz, H-8), 5.45 (1H, d, *J* = 9.8 Hz, CH_3CONH), 5.39 (1H, dd, *J* = 8.6 Hz, *J* = 2.2 Hz, H-7), 5.23 (1H, ddd, *J* = 11.8 Hz, *J* = 10.2 Hz, *J* = 4.5 Hz, H-4), 4.38 (1H, ddd, *J* = 10.8 Hz, *J* = 2.2 Hz, H-6), 4.25 (1H, dd, *J* = 12.5 Hz, *J* = 2.9 Hz, H-9a), 4.18-4.08 (2H, m, H-5, H9-b), 3.79 (3H, s, CO_2CH_3), 3.48 (1H, dd, *J* = 13.3 Hz, *J* = 4.5 Hz, H-3_{eq}), 2.71 (1H, dd, *J* = 13.3 Hz, *J* = 11.9 Hz, H-3_{ax}), 2.17 (3H, s, CH_3CO), 2.10 (3H, s, CH_3CO), 2.07 (3H, s, CH_3CO), 2.06 (3H, s, CH_3CO), 1.91 (3H, s, CH_3CONH). ^{13}C NMR (101 MHz, $CDCl_3$) δ 170.9, 170.7, 170.4, 170.3, 170.1 (4x $CH_3\underline{C}O$, C-1), 166.5 ($CH_3\underline{C}ONH$), 149.9 ($\underline{C}$CH-triazole), 149.8 (Py-$\underline{C}$H), 148.9 ($C_{quat.}$), 137.0 (Py-$\underline{C}$H), 123.2 (Py-$\underline{C}$H), 121.1 (Py-$\underline{C}$H), 120.7 (triazole-$\underline{C}$H), 88.7 (C-2), 73.9 (C-6), 68.6 (C-4), 68.3 (C-8), 67.0 (C-7), 62.3 (C-9), 54.2 ($CO_2\underline{C}H_3$), 49.5 (C-5), 36.5 (C-3), 23.3 ($\underline{C}H_3CONH$), 21.3, 21.0, 20.9, 20.9 (4x $\underline{C}H_3CO$). HRMS (ESI): *m/z* calculated for $C_{27}H_{33}N_5NaO_{12}$ $[M+Na]^+$: 642.2023; found: 642.2014.

Methyl 4,7,8,9-tetra-*O*-acetyl-2-{4-[(*N*-methylbenzylamino)methyl]-1*H*-1,2,3-triazol-1-yl}-5-acetamido-2,3,5-trideoxy-D-*glycero*-α-D-*galacto*non-2-ulopyranosonate (**2e**)

Yield: 71% (26 mg; 0.038 mmol). ^1H NMR (400 MHz, $CDCl_3$) δ 7.89 (1H, s, C*H*-triazole), 7.39-7.21 (5H, m, H-Ph), 5.45 (1H, ddd, *J* = 8.4 Hz, *J* = 5.6 Hz, *J* = 2.7 Hz, H-8), 5.42-5.35 (2H, m, CH_3CONH, H-7), 5.18 (1H, ddd, *J* = 12.0 Hz, *J* = 10.3 Hz, *J* = 4.5 Hz, H-4), 4.35-4.25 (2H, m, H-6, H-9a), 4.16-4.04 (2H, m, H-5, H-9b), 3.76 (3H, s, CO_2CH_3), 3.74 (2H, s, CH_2), 3.57 (2H, s, CH_2), 3.44 (1H, dd, *J* = 13.3 Hz, *J* = 4.5 Hz, H-3_{eq}), 2.68 (1H, dd, *J* = 13.3 Hz, *J* = 12.0 Hz, H-3_{ax}), 2.25 (3H, s, NCH_3), 2.17 (3H, s, CH_3CO), 2.11 (3H, s, CH_3CO), 2.07 (3H, s, CH_3CO), 2.04 (3H, s, CH_3CO), 1.91 (3H, s, CH_3CONH). ^{13}C NMR (101 MHz, $CDCl_3$) δ 171.0, 170.7, 170.4, 170.3, 170.2 (4x $CH_3\underline{C}O$, C-1), 166.7 ($CH_3\underline{C}ONH$), 145.9 ($\underline{C}$CH-triazole), 138.7 ($C_{quat.}$), 129.3 (2x Ph-$\underline{C}$H), 128.4 (2x Ph-$\underline{C}$H), 127.2 (Ph-$\underline{C}$H), 121.8 (triazole-$\underline{C}$H), 88.5 (C-2), 73.9 (C-6), 68.7 (C-4), 68.2 (C-8), 67.1 (C-7), 62.4 (C-9), 61.4 ($\underline{C}H_2$), 54.1 ($CO_2\underline{C}H_3$), 51.9 ($\underline{C}H_2$), 49.4 (C-5), 42.2 ($N\underline{C}H_3$), 36.2 (C-3), 23.3 ($\underline{C}H_3CONH$), 21.3, 21.0, 20.9, 20.8 (4x $\underline{C}H_3CO$). HRMS (ESI): *m/z* calculated for $C_{31}H_{42}N_5O_{12}$ $[M+H]^+$: 676.2830; found: 676.2813.

Methyl 4,7,8,9-tetra-*O*-acetyl-2-[4-(1-hydroxypropyl)-1*H*-1,2,3-triazol-1-yl]-5-acetamido-2,3,5-trideoxy-D-*glycero*-α-D-*galacto*non-2-ulopyranosonate (**2f**)

Yield: 54% (24 mg; 0.040 mmol). ^1H NMR (400 MHz, $CDCl_3$) δ 7.87 (1H, d, *J* = 4.3 Hz, C*H*-triazole), 5.57 (1H, d, *J* = 9.8 Hz, CH_3CONH), 5.44-5.34 (2H, H-8, H-7), 5.16 (1H, ddd, *J* = 11.9 Hz, *J* = 10.3 Hz, *J* = 4.4 Hz, H-4), 4.84-4.78 (1H, m, C*H*OH), 4.37-4.28 (2H, m, H-6, H-9a), 4.12 (1H, q, *J* = 10.3 Hz, H-5), 4.02 (1H, ddd, *J* = 12.5 Hz, *J* = 5.7 Hz, *J* = 1.8 Hz, H9-b), 3.77 (3H, s, CO_2CH_3), 3.43 (1H, dd, *J* = 13.3 Hz, *J* = 4.5 Hz, H-3_{eq}), 2.78 (1H, d, *J* = 19.5 Hz, O*H*), 2.66 (1H, dd, *J* = 13.3 Hz, *J* = 12.0 Hz, H-3_{ax}), 2.15 (3H, s, CH_3CO), 2.10 (3H, s, CH_3CO), 2.06 (3H, s, CH_3CO), 2.04 (3H, s, CH_3CO), 1.98-1.91 (2H, m, CH_2CH_3), 1.89 (3H, s, CH_3CONH), 1.01 (3H, td, *J* = 7.4 Hz, *J* = 1.1 Hz, CH_2CH_3). ^{13}C NMR (101 MHz, $CDCl_3$) δ 171.0, 170.8, 170.5, 170.4,

170.2 (4x $CH_3\underline{C}O$, C-1), 166.5 ($CH_3\underline{C}ONH$), 152.0 ($\underline{C}CH$-triazole), 119.8 (triazole-$\underline{C}H$), 88.6 (C-2), 74.1 (C-6), 68.6 (C-4, C-8), 68.3 (CHOH), 67.2 (C-7), 62.5 (C-9), 54.2 ($CO_2\underline{C}H_3$), 49.3 (C-5), 36.1 (C-3), 30.1 ($\underline{C}H_2CH_3$), 23.2 ($\underline{C}H_3CONH$), 21.3, 21.0, 20.9, 20.9 (4x $\underline{C}H_3CO$), 9.9 ($CH_2\underline{C}H_3$). HRMS (ESI): *m/z* calculated for $C_{25}H_{36}N_4NaO_{13}$ $[M+Na]^+$: 236.2177; found: 236.2166.

Methyl 4,7,8,9-tetra-*O*-acetyl-2-[4-(2-hydroxypropyl)-1*H*-1,2,3-triazol-1-yl]-5-acetamido-2,3,5-trideoxy-D-*glycero*-α-D-*galacto*non-2-ulopyranosonate (**2g**)

Yield: 45% (20 mg; 0.033 mmol). ^{1}H NMR (400 MHz, $CDCl_3$) δ 7.78 (1H, d, *J* = 3.8 Hz, C*H*-triazole), 5.46-5.35 (3H, m, H-8, $CH_3CON\mathit{H}$, H-7), 5.16 (1H, ddd, *J* = 12.0 Hz, *J* = 10.3 Hz, *J* = 4.4 Hz, H-4), 4.34-4.28 (2H, m, H-6, H-9a), 4.21-4.14 (1H, m, C*H*OH), 4.11 (1H, q, *J* = 10.4 Hz, H-5), 4.04 (1H, ddd, *J* = 12.5 Hz, *J* = 5.8 Hz, *J* = 1.9 Hz, H9-b), 3.78 (3H, s, $CO_2C\mathit{H}_3$), 3.43 (1H, dd, *J* = 13.2 Hz, *J* = 4.5 Hz, H-3_{eq}), 3.05 (1H, s, O*H*), 2.91 (1H, dd, *J* = 15.1 Hz, *J* = 3.5 Hz, $C\mathit{H}_2CHOH$), 2.79 (1H, ddd, *J* = 15.4 Hz, *J* = 8.5 Hz, *J* = 2.5 Hz, $C\mathit{H}_2CHOH$), 2.67 (1H, dd, *J* = 13.3 Hz, *J* = 12.1 Hz, H-3_{ax}), 2.17 (3H, s, $C\mathit{H}_3CO$), 2.12 (3H, s, $C\mathit{H}_3CO$), 2.07 (3H, s, $C\mathit{H}_3CO$), 2.05 (3H, s, $C\mathit{H}_3CO$), 1.90 (3H, s, $C\mathit{H}_3CONH$), 1.29 (3H, dd, *J* = 6.2 Hz, *J* = 1.4 Hz, $C\mathit{H}_3CHOH$). ^{13}C NMR (101 MHz, $CDCl_3$) δ 171.0, 170.8, 170.5, 170.4, 170.2 (4x $CH_3\underline{C}O$, C-1), 166.6 ($CH_3\underline{C}ONH$), 146.2 ($\underline{C}CH$-triazole), 120.9 (triazole-$\underline{C}H$), 88.5 (C-2), 74.0 (C-6), 68.6 (C-4), 68.4 (C-8), 67.1 (C-7, CHOH), 62.5 (C-9), 54.2 ($CO_2\underline{C}H_3$), 49.4 (C-5), 36.1 (C-3), 35.0 ($\underline{C}H_2CHOH$), 23.3 ($\underline{C}H_3CONH$), 23.0 ($\underline{C}H_3CHOH$), 21.3, 21.0, 20.9, 20.9 (4x $\underline{C}H_3CO$). HRMS (ESI): *m/z* calculated for $C_{25}H_{36}N_4NaO_{13}$ $[M+Na]^+$: 236.2177; found: 236.2166.

Methyl 4,7,8,9-tetra-*O*-acetyl-2-(4-phenylmethyl-1*H*-1,2,3-triazol-1-yl)-5-acetamido-2,3,5-trideoxy-D-*glycero*-α-D-*galacto*non-2-ulopyranosonate (**2h**)

Yield: 49% (24 mg; 0.038 mmol). ^{1}H NMR (400 MHz, $CDCl_3$) δ 7.71 (1H, s, C*H*-triazole), 7.34-7.28 (4H, m, H-Ph), 7.24-7.19 (1H, m, H-Ph), 5.44 (1H, d, *J* = 9.8 Hz, $CH_3CON\mathit{H}$), 5.40 (1H, ddd, *J* = 8.3 Hz, *J* = 5.6 Hz, *J* = 2.6 Hz, H-8), 5.35 (1H, dd, *J* = 8.5 Hz, *J* = 2.2 Hz, H-7), 5.15 (1H, ddd, *J* = 11.9 Hz, *J* = 10.2 Hz, *J* = 4.5 Hz, H-4), 4.31-4.24 (2H, m, H-6, H-9a), 4.14-4.02 (4H, m, H-5, H-9b, $C\mathit{H}_2$), 3.76 (3H, s, $CO_2C\mathit{H}_3$), 3.42 (1H, dd, *J* = 13.3 Hz, *J* = 4.5 Hz, H-3_{eq}), 2.65 (1H, dd, *J* = 13.3 Hz, *J* = 12.0 Hz, H-3_{ax}), 2.13 (3H, s, $C\mathit{H}_3CO$), 2.09 (3H, s, $C\mathit{H}_3CO$), 2.06 (3H, s, $C\mathit{H}_3CO$), 2.04 (3H, s, $C\mathit{H}_3CO$), 1.89 (3H, s, $C\mathit{H}_3CONH$). ^{13}C NMR (101 MHz, $CDCl_3$) δ 170.9, 170.7, 170.4, 170.3, 170.2 (4x $CH_3\underline{C}O$, C-1), 166.6 ($CH_3\underline{C}ONH$), 147.8 ($\underline{C}CH$-triazole), 138.8 ($C_{quat.}$), 128.9 (2x Ph-$\underline{C}H$), 128.7 (2x Ph-$\underline{C}H$), 126.6 (Ph-$\underline{C}H$), 120.5 (triazole-$\underline{C}H$), 88.5 (C-2), 73.9 (C-6), 68.7 (C-4), 68.3 (C-8), 67.1 (C-7), 62.4 (C-9), 54.1 ($CO_2\underline{C}H_3$), 49.4 (C-5), 36.1 (C-3), 32.1 ($\underline{C}H_2$), 23.3 ($\underline{C}H_3CONH$), 21.3, 21.0, 20.9, 20.8 (4x $\underline{C}H_3CO$). HRMS (ESI): *m/z* calculated for $C_{29}H_{36}N_4NaO_{12}$ $[M+Na]^+$: 655.2227; found: 655.2215.

Compounds **3a–h**:

5-Acetamido-3,5-dideoxy-2-{4-[2-(trifluoromethyl)phenyl]-1*H*-1,2,3-triazol-1-yl}-D-*glycero*-α-D-*galacto*-non-2-ulopyranosidic acid (**3a**)

Yield: 85% (20 mg; 0.040 mmol); $[\alpha]_D^{20}$ -26.6 (c 1, H_2O). ^1H NMR (400 MHz, D_2O) δ 8.33 (1H, s, C*H*-triazole), 7.89 (1H, d, *J* = 7.8 Hz, H-Ph), 7.73 (1H, t, *J* = 7.6 Hz, H-Ph), 7.69-7.60 (2H, m, H-Ph), 4.10-3.84 (5H, ms - overlapped signals, H-4, H-5, H-6, H-7, H-9a), 3.70-3.61 (2H, m, H-8, H-9b), 3.39-3.30 (1H, m, H-3_{eq}), 2.35 (1H, t, *J* = 11.4 Hz, H-3_{ax}), 2.08 (3H, s, CH_3CONH). ^{13}C NMR (101 MHz, D_2O) δ 175.0 (C-1), 170.5 (CH_3CONH), 144.5 (CCH-triazole), 132.3 (Ph-CH), 132.0 (Ph-CH), 129.4 (Ph-CH), 127.8 ($C_{quat.}$), 126.4 (Ph-CH), 122.5 (CH-triazole), 91.0 (C-2), 74.2, 71.4, 68.1, 68.0, (C-4, C-6, C-7, C-8), 62.6 (C-9), 51.5 (C-5), 39.5 (C-3), 22.1 (CH_3CONH). HRMS (ESI): *m/z* calculated for $C_{20}H_{23}F_3N_4NaO_8$ $[M+Na]^+$: 527.1360; found: 527.1352.

5-Acetamido-3,5-dideoxy-2-{4-[3-(trifluoromethyl)phenyl]-1*H*-1,2,3-triazol-1-yl}-D-*glycero*-α-D-*galacto*-non-2-ulopyranosidic acid (**3b**)

Yield: 98% (23 mg; 0.046 mmol); $[\alpha]_D^{20}$ -20.0 (c 1, H_2O). ^1H NMR (400 MHz, D_2O) δ 8.51 (1H, s, C*H*-triazole), 7.96 (1H, s, H-Ph), 7.90 (1H, d, *J* = 7.8 Hz, H-Ph), 7.61 (1H, d, *J* = 7.9 Hz, H-Ph), 7.53 (1H, d, *J* = 7.8 Hz, H-Ph), 4.11-3.88 (5H, ms - overlapped signals, H-4, H-5, H-6, H-7, H-9a), 3.72-3.63 (2H, m, H-8, H-9b), 3.38-3.29 (1H, m, H-3_{eq}), 2.29 (1H, t, *J* = 11.5 Hz, H-3_{ax}), 2.09 (3H, s, CH_3CONH). ^{13}C NMR (101 MHz, D_2O) δ 175.0 (C-1), 170.4 (CH_3CONH), 145.9 (CCH-triazole), 129.9 ($C_{quat.}$), 129.6 (Ph-CH), 129.0 (Ph-CH), 125.1 (Ph-CH), 122.2 (Ph-CH), 120.2 (CH-triazole), 91.0 (C-2), 74.2, 71.3, 68.1, 68.0, (C-4, C-6, C-7, C-8), 62.7 (C-9), 51.6 (C-5), 39.8 (C-3), 22.0 (CH_3CONH). HRMS (ESI): *m/z* calculated for $C_{20}H_{23}F_3N_4NaO_8$ $[M+Na]^+$: 527.1360; found: 527.1346.

5-Acetamido-3,5-dideoxy-2-{4-[4-(trifluoromethyl)phenyl]-1*H*-1,2,3-triazol-1-yl}-D-*glycero*-α-D-*galacto*-non-2-ulopyranosidic acid (**3c**)

Yield: 69% (14 mg; 0.028 mmol); $[\alpha]_D^{20}$ -18.3 (c 1, H_2O). ^1H NMR (400 MHz, D_2O) δ 8.58 (1H, s, C*H*-triazole), 7.90 (2H, d, *J* = 8.1 Hz, H-Ph), 7.76 (2H, d, *J* = 8.2 Hz, H-Ph), 4.11-3.84 (5H, ms - overlapped signals, H-4, H-5, H-6, H-7, H-9a), 3.71-3.64 (2H, m, H-8, H-9b), 3.33 (1H, dd, *J* = 12.5 Hz, *J* = 3.9 Hz, H-3_{eq}), 2.30 (1H, t, *J* = 11.5 Hz, H-3_{ax}), 2.08 (3H, s, CH_3CONH). ^{13}C NMR (101 MHz, D_2O) δ 175.0 (C-1), 170.5 (CH_3CONH), 146.0 (CCH-triazole), 132.9 ($C_{quat.}$), 129.8 ($C_{quat.}$), 126.0 (4x Ph-CH), 120.6 (CH-triazole), 91.0 (C-2), 74.2, 71.3, 68.1, 68.0, (C-4, C-6, C-7, C-8), 62.7 (C-9), 51.5 (C-5), 39.8 (C-3), 22.0 (CH_3CONH). HRMS (ESI): *m/z* calculated for $C_{20}H_{23}F_3N_4NaO_8$ $[M+Na]^+$: 527.1360; found: 527.1351.

5-Acetamido-3,5-dideoxy-2-[4-(pyridine-2-yl)-1*H*-1,2,3-triazol-1-yl]-D-*glycero*-α-D-*galacto*-non-2-ulopyranosidic acid (**3d**)

Yield: 71% (17 mg; 0.040 mmol); $[\alpha]_D^{20}$ -32.0 (c 1, H_2O). ^1H NMR (400 MHz, D_2O) δ 8.57 (1H, s, C*H*-triazole), 8.50 (1H, s, H-Py), 7.90 (2H, d, *J* = 4.3 Hz, H-Py), 7.39 (1H, q, *J* = 4.6 Hz, H-Py), 4.09-3.88 (5H, ms - overlapped signals, H-4, H-5, H-6, H-7, H-9a), 3.71-3.64 (2H, m, H-8, H-9b), 3.36 (1H, dd, *J* = 12.5 Hz, *J* = 4.0 Hz, $H\text{-}3_{eq}$), 2.30 (1H, t, *J* = 11.5 Hz, $H\text{-}3_{ax}$), 2.08 (3H, s, CH_3CONH). ^{13}C NMR (101 MHz, D_2O) δ 175.0 (C-1), 170.5 ($CH_3\underline{C}ONH$), 149.0 (Py-C̲H), 146.6 (C̲CH-triazole), 138.4 (Py-C̲H), 124.0 (Py-C̲H), 121.3 (Py-C̲H), 121.1 (C̲H-triazole), 91.0 (C-2), 74.2, 71.3, 68.1, 68.0, (C-4, C-6, C-7, C-8), 62.7 (C-9), 51.5 (C-5), 39.7 (C-3), 22.1 ($\underline{C}H_3CONH$). HRMS (ESI): *m/z* calculated for $C_{18}H_{24}N_5O_8$ $[M+H]^+$: 438.1619; found: 438.1623.

5-Acetamido-3,5-dideoxy-2-{4-[(*N*-methylbenzylamino)methyl]-1*H*-1,2,3-triazol-1-yl}-D-*glycero*-α-D-*galacto*-non-2-ulopyranosidic acid (**3e**)

Yield: 82% (18 mg; 0.037 mmol); $[\alpha]_D^{20}$ -18.1 (c 1, H_2O). ^1H NMR (400 MHz, D_2O) δ 8.18 (1H, s, C*H*-triazole), 7.48-7.42 (3H, m, H-Ph), 7.41-7.37 (2H, m, H-Ph), 4.06-3.83 (9H, ms - overlapped signals, H-4, H-5, H-6, H-7, H-9a, 2x CH_2), 3.69-3.60 (2H, m, H-8, H-9b), 3.28 (1H, dd, *J* = 12.6 Hz, *J* = 3.8 Hz, $H\text{-}3_{eq}$), 2.42 (3H, s, NCH_3), 2.24 (1H, t, *J* = 11.5 Hz, $H\text{-}3_{ax}$), 2.07 (3H, s, CH_3CONH). ^{13}C NMR (101 MHz, D_2O) δ 175.0 (C-1), 170.5 ($CH_3\underline{C}ONH$), 140.6 (C̲CH-triazole), 130.3 (2x Ph-C̲H), 128.8 (2x Ph-C̲H), 128.6 (Ph-C̲H), 123.6 (C̲H-triazole), 90.9 (C-2), 74.1, 71.3, 68.0, 67.9, (C-4, C-6, C-7, C-8), 62.7 (C-9), 60.0 ($\underline{C}H_2$), 51.5 (C-5), 49.7 ($\underline{C}H_2$), 40.3 ($N\underline{C}H_3$), 39.6 (C-3), 22.0 ($\underline{C}H_3CONH$). HRMS (ESI): *m/z* calculated for $C_{22}H_{32}N_5O_8$ $[M+H]^+$: 494.2245; found: 494.2249.

5-Acetamido-3,5-dideoxy-2-[4-(1-hydroxypropyl)-1*H*-1,2,3-triazol-1-yl]-D-*glycero*-α-D-*galacto*-non-2-ulopyranosidic acid (**3f**)

Yield: 100% (20 mg; 0.048 mmol); $[\alpha]_D^{20}$ -24.7 (c 1, H_2O). ^1H NMR (400 MHz, D_2O) δ 8.14 (1H, s, C*H*-triazole), 4.84 (1H, t, *J* = 6.9 Hz, C*H*OH), 4.06-3.83 (5H, ms - overlapped signals, H-4, H-5, H-6, H-7, H-9a), 3.69-3.60 (2H, m, H-8, H-9b), 3.27 (1H, dd, *J* = 12.7 Hz, *J* = 3.9 Hz, $H\text{-}3_{eq}$), 2.26 (1H, t, *J* = 11.0 Hz, $H\text{-}3_{ax}$), 2.07 (3H, s, CH_3CONH), 1.95-1.85 (2H, m, CH_2CH_3), 0.90 (3H, t, *J* = 7.4 Hz, CH_2CH_3). ^{13}C NMR (101 MHz, D_2O) δ 175.0 (C-1), 170.6 ($CH_3\underline{C}ONH$), 150.1 (C̲CH-triazole), 120.6 (C̲H-triazole), 90.8 (C-2), 74.1, 71.3, 68.1, 68.0, (C-4, C-6, C-7, C-8), 67.4 (C̲HOH), 62.7 (C-9), 51.5 (C-5), 39.6 (C-3), 29.0 ($\underline{C}H_2CH_3$), 22.0 ($\underline{C}H_3CONH$) 9.0 ($CH_2\underline{C}H_3$). HRMS (ESI): *m/z* calculated for $C_{16}H_{26}N_4NaO_9$ $[M+Na]^+$: 441.1592; found: 441.1590.

5-Acetamido-3,5-dideoxy-2-[4-(2-hydroxypropyl)-1*H*-1,2,3-triazol-1-yl]-D-*glycero*-α-D-*galacto*-non-2-ulopyranosidic acid (**3g**)

Yield: 94% (16 mg; 0.038 mmol); $[\alpha]_D^{20}$ -34.5 (c 1, H_2O). ^1H NMR (400 MHz, D_2O) δ 8.03 (1H, s, C*H*-triazole), 4.13 (1H, h, *J* = 6.4 Hz, C*H*OH), 4.05-3.83 (5H, ms - overlapped signals, H-4, H-5, H-6, H-7, H-9a), 3.69-3.60 (2H, m, H-8, H-9b), 3.25 (1H, dd, *J* = 12.6 Hz, *J* = 4.0 Hz, H-3_{eq}), 2.96-2.83 (2H, m, CH_2CHOH), 2.27 (1H, t, *J* = 11.5 Hz, H-3_{ax}), 2.07 (3H, s, CH_3CONH), 1.21 (3H, d, *J* = 6.2 Hz, CH_3CHOH). ^{13}C NMR (101 MHz, D_2O) δ 175.0 (C-1), 170.8 ($CH_3\underline{C}ONH$), 144.5 ($\underline{C}$CH-triazole), 121.7 ($\underline{C}$H-triazole), 90.7 (C-2), 74.0, 71.3, 68.1, 68.0, (C-4, C-6, C-7, C-8), 66.9 ($\underline{C}$HOH), 62.7 (C-9), 51.5 (C-5), 39.6 (C-3), 33.9 (CH_2CHOH), 22.0 ($\underline{C}H_3$CONH) 21.4 ($CH_3\underline{C}HOH$). HRMS (ESI): *m/z* calculated for $C_{16}H_{26}N_4NaO_9$ $[M+Na]^+$: 441.1592; found: 441.1592.

5-Acetamido-3,5-dideoxy-2-(4-phenylmethyl-1*H*-1,2,3-triazol-1-yl)-D-*glycero*-α-D-*galacto*-non-2-ulopyranosidic acid (**3h**)

Yield: 74% (15 mg; 0.033 mmol); $[\alpha]_D^{20}$ -38.4 (c 1, H_2O). ^1H NMR (400 MHz, D_2O) δ 7.99 (1H, s, C*H*-triazole), 7.41-7.34 (2H, m, H-Ph), 7.34-7.27 (3H, m, H-Ph), 4.09 (2H, s, CH_2), 4.03-3.81 (5H, ms - overlapped signals, H-4, H-5, H-6, H-7, H-9a), 3.67-3.58 (2H, m, H-8, H-9b), 3.23 (1H, dd, *J* = 12.5 Hz, *J* = 3.9 Hz, H-3_{eq}), 2.21 (1H, t, *J* = 11.8 Hz, H-3_{ax}), 2.06 (3H, s, CH_3CONH). ^{13}C NMR (101 MHz, D_2O) δ 175.0 (C-1), 170.7 ($CH_3\underline{C}ONH$), 147.3 ($\underline{C}$CH-triazole), 139.0 ($C_{quat.}$), 128.9 (2x Ph-$\underline{C}$H), 128.6 (2x Ph-$\underline{C}$H), 126.7 (Ph-$\underline{C}$H), 121.1 ($\underline{C}$H-triazole), 90.7 (C-2), 74.0, 71.2, 68.1, 68.0, (C-4, C-6, C-7, C-8), 62.7 (C-9), 51.5 (C-5), 39.7 (C-3), 30.80 ($\underline{C}H_2$), 22.0 ($\underline{C}H_3$CONH). HRMS (ESI): *m/z* calculated for $C_{20}H_{26}N_4NaO_8$ $[M+Na]^+$: 473.1643; found: 473.1638.

^{1}H NMR (400 MHz, $CDCl_3$) spectrum of compound **1**

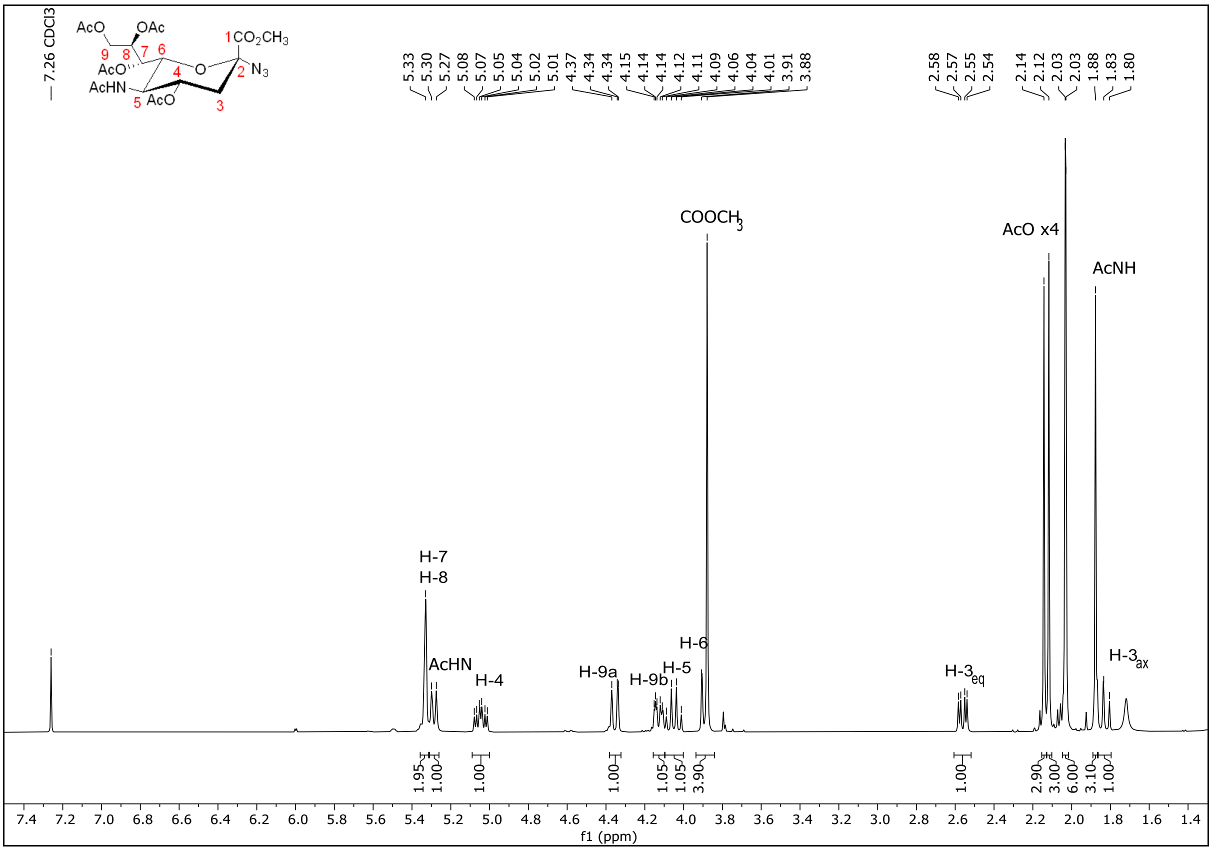

^{13}C NMR (101 MHz, $CDCl_3$) spectrum of compound **1**

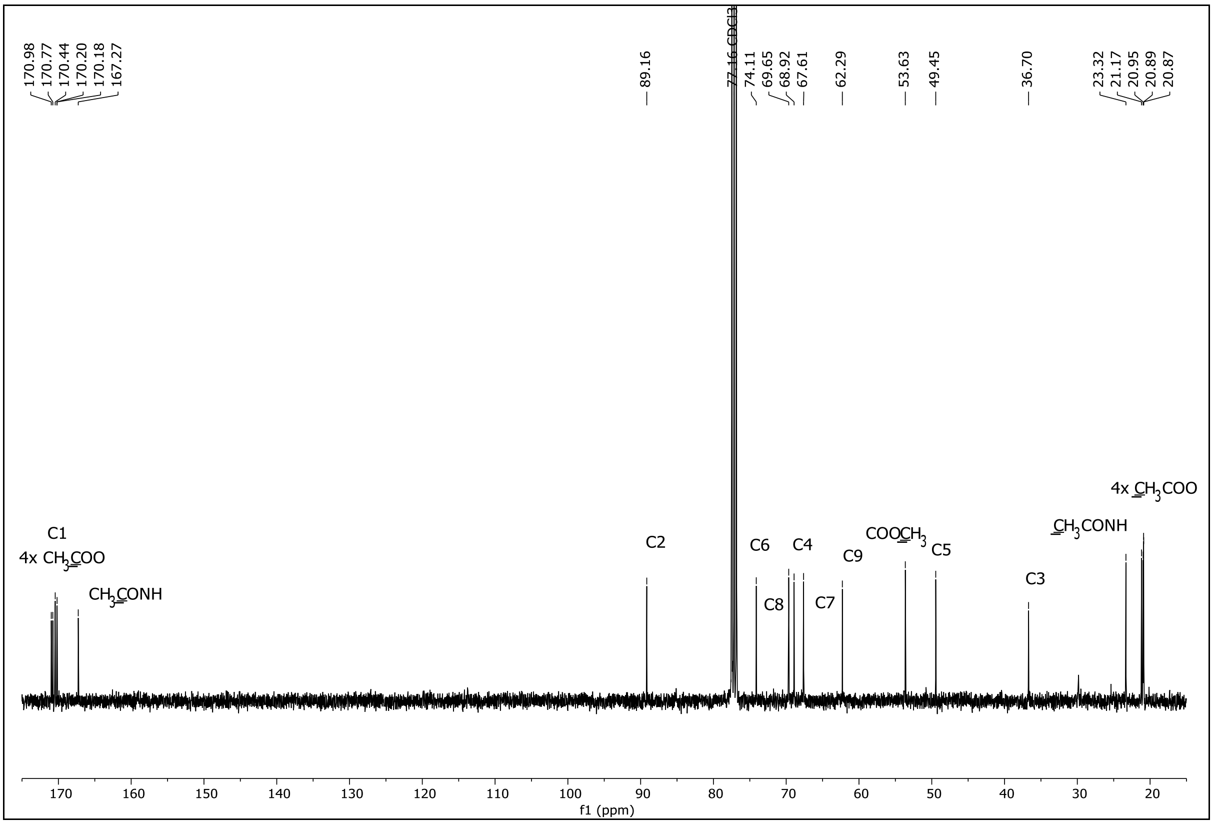

^{1}H NMR (400 MHz, $CDCl_3$) spectrum of compound **2a**

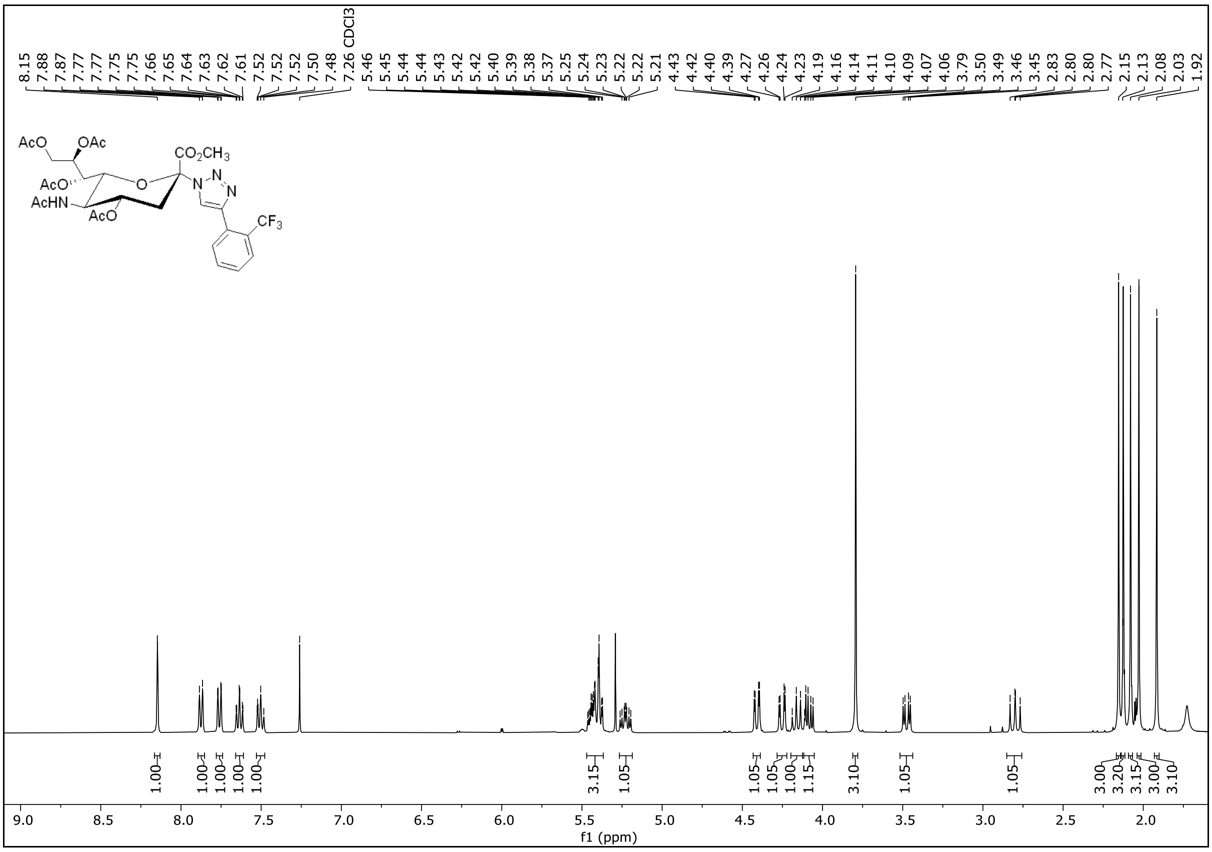

^{13}C NMR (101 MHz, $CDCl_3$) spectrum of compound **2a**

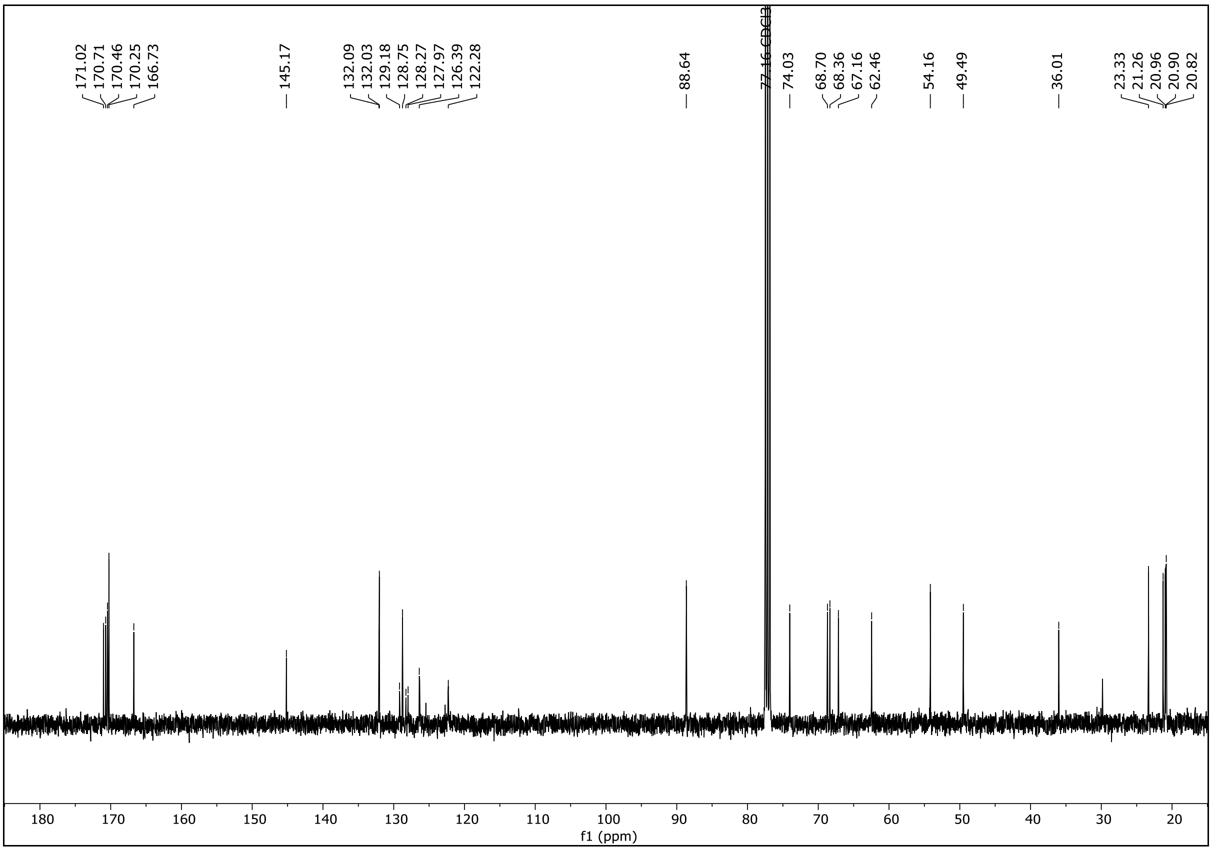

^{1}H NMR (400 MHz, $CDCl_3$) spectrum of compound **2b**

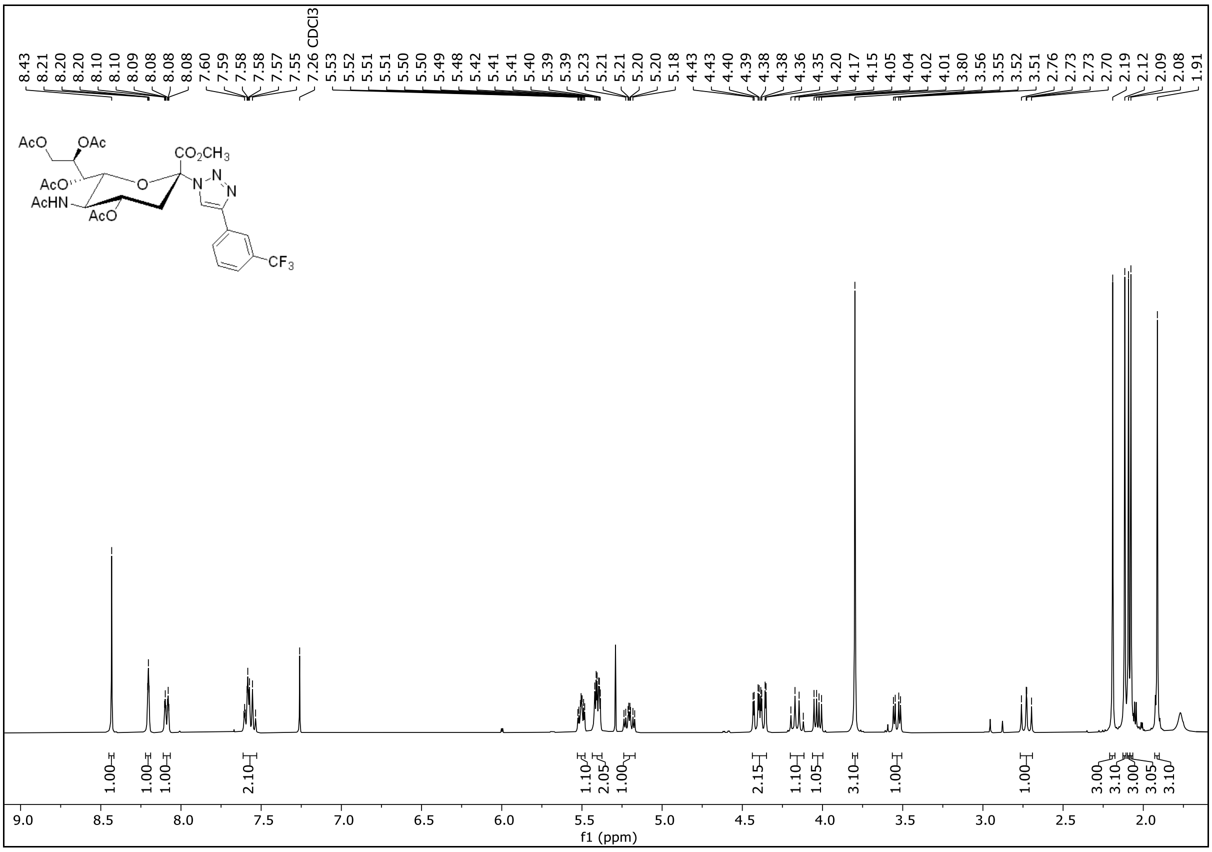

^{13}C NMR (101 MHz, $CDCl_3$) spectrum of compound **2b**

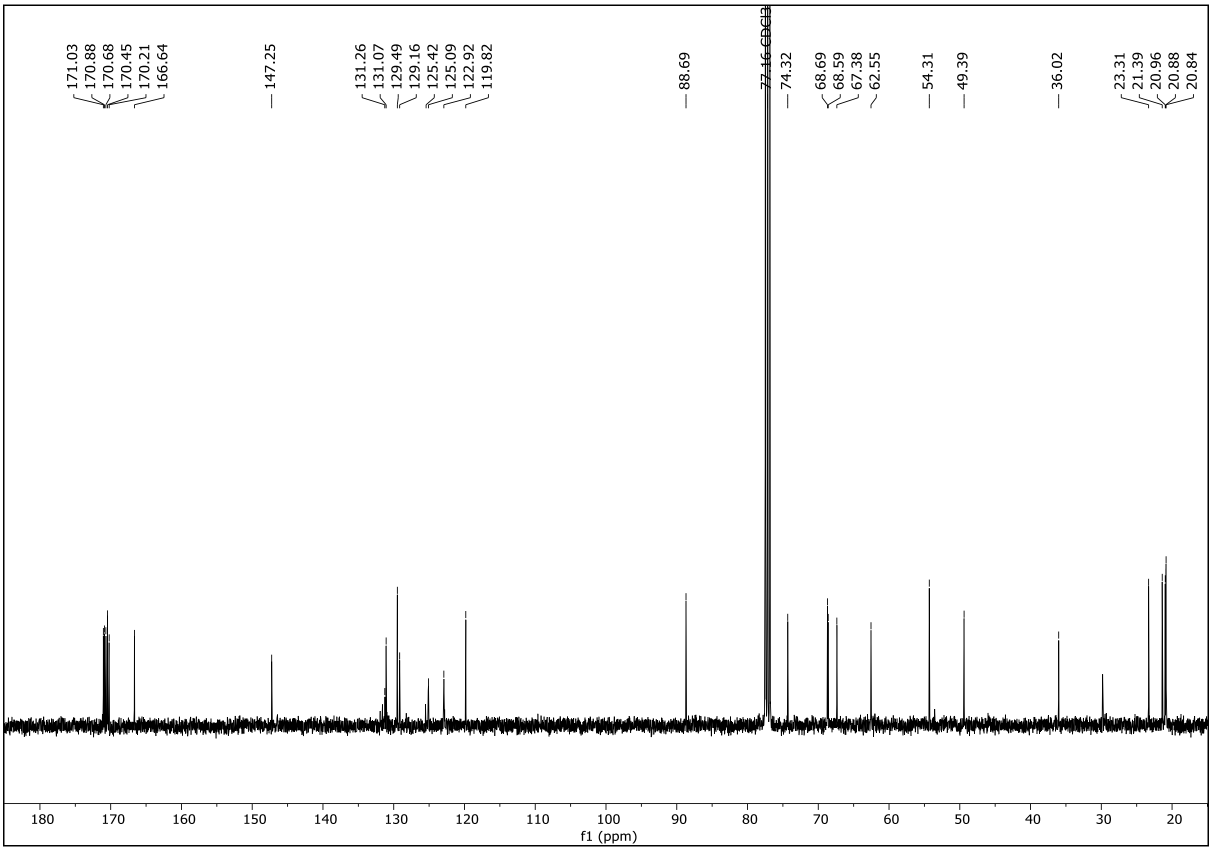

^{1}H NMR (400 MHz, $CDCl_3$) spectrum of compound **2c**

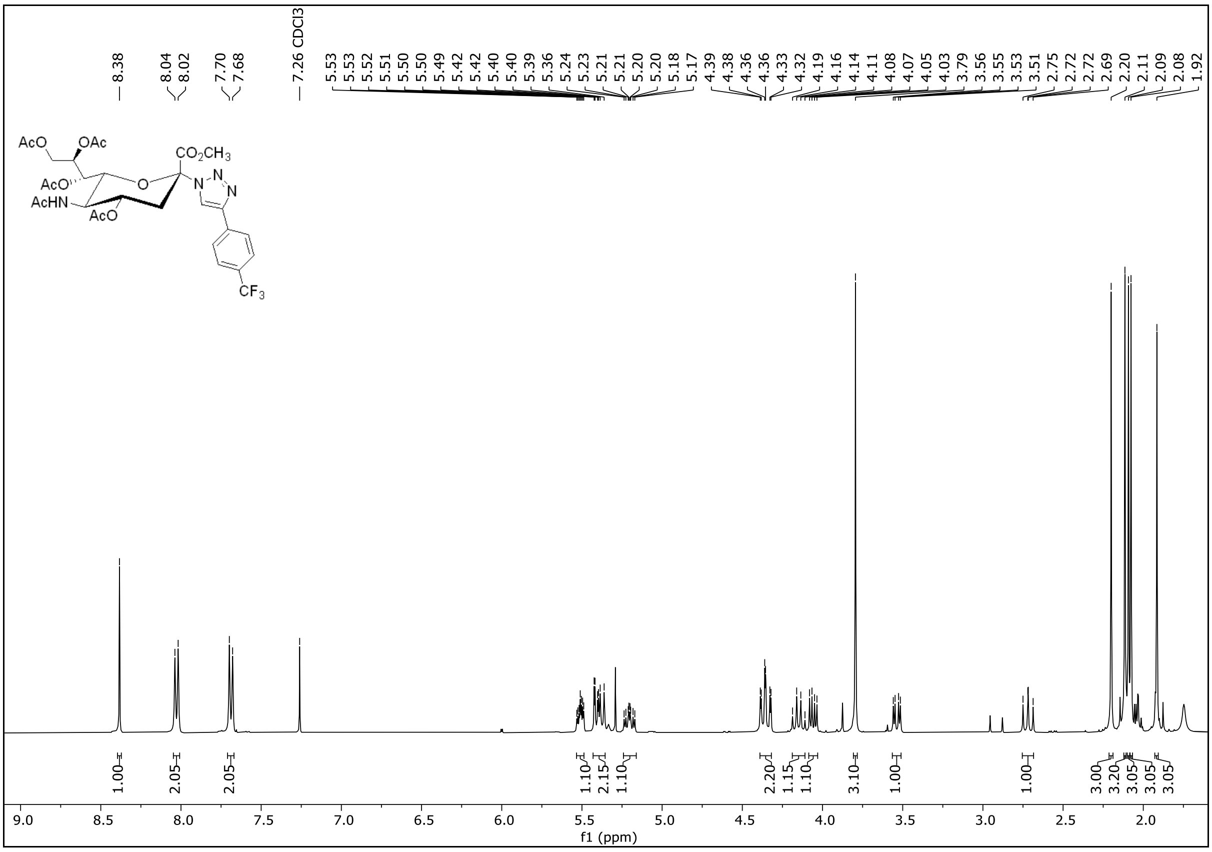

^{13}C NMR (101 MHz, $CDCl_3$) spectrum of compound **2c**

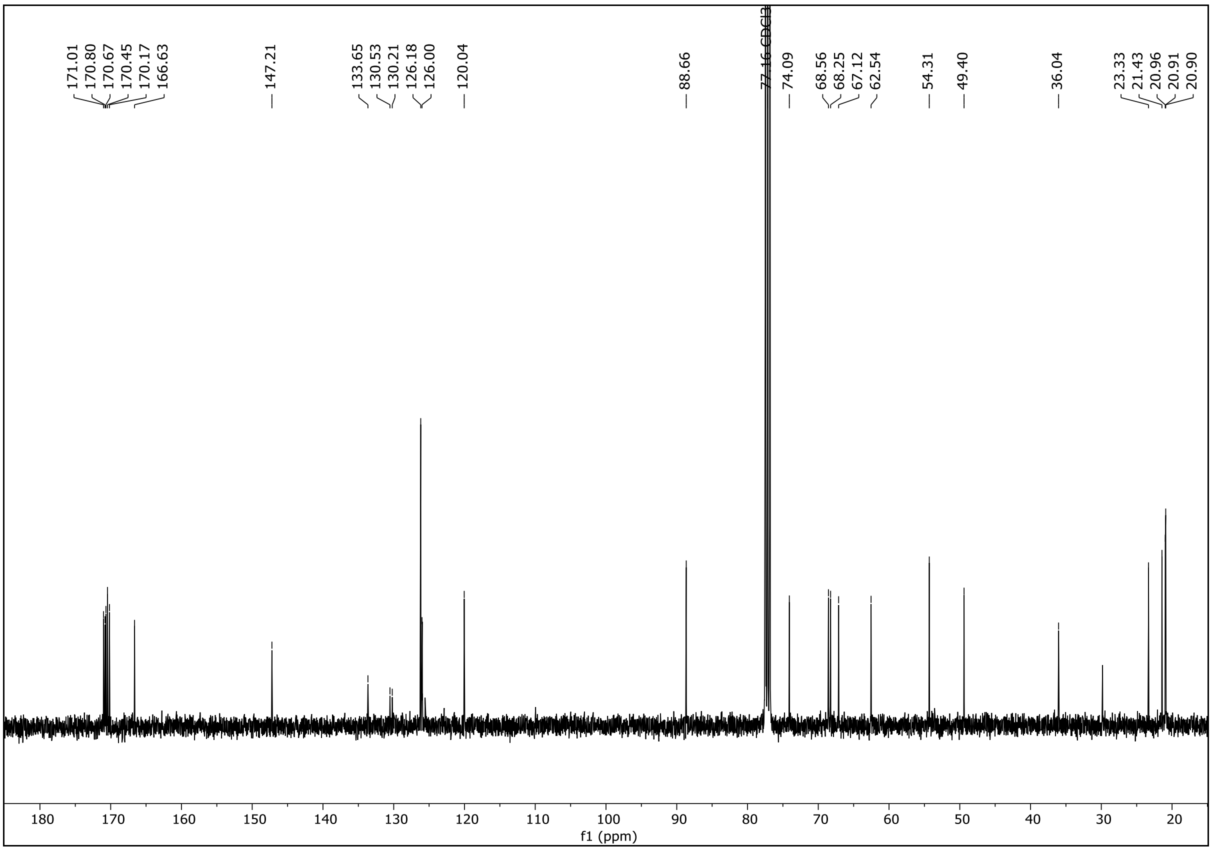

^{1}H NMR (400 MHz, $CDCl_3$) spectrum of compound **2d**

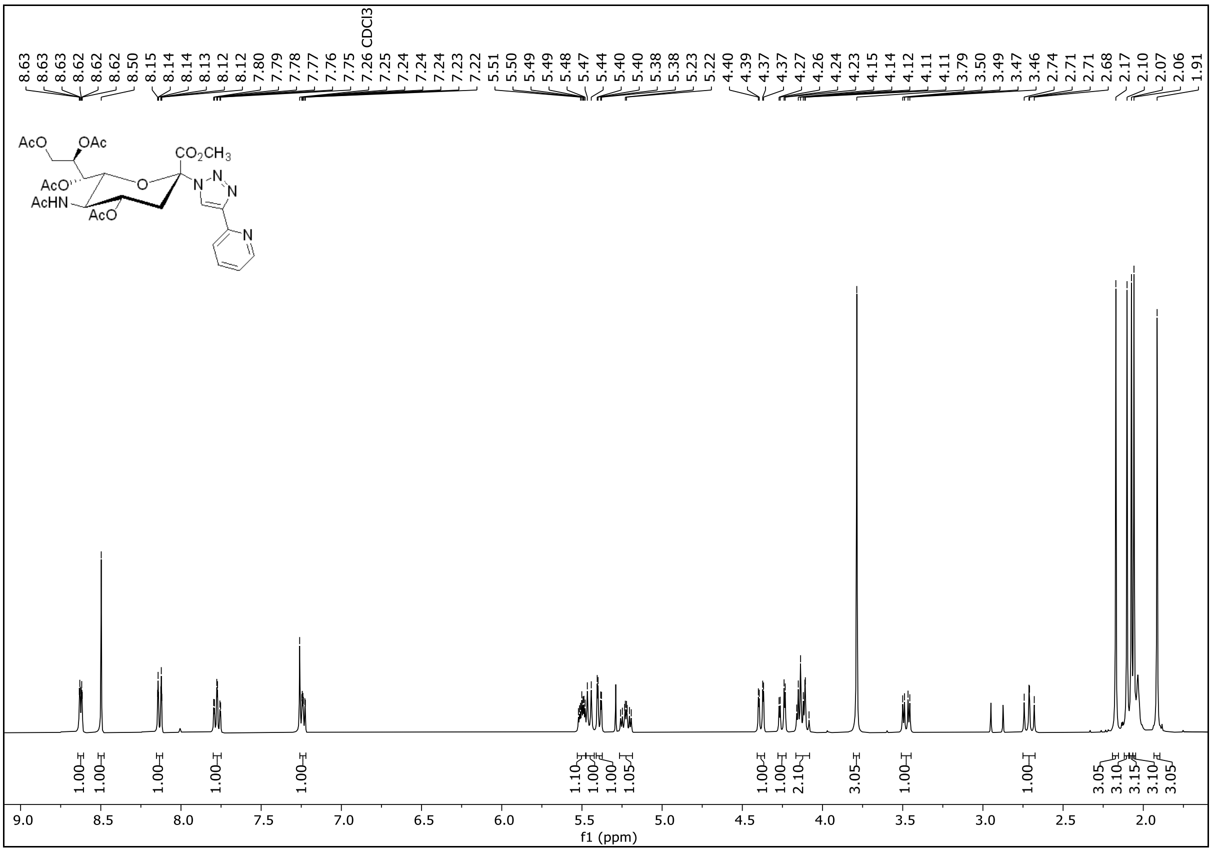

^{13}C NMR (101 MHz, $CDCl_3$) spectrum of compound **2d**

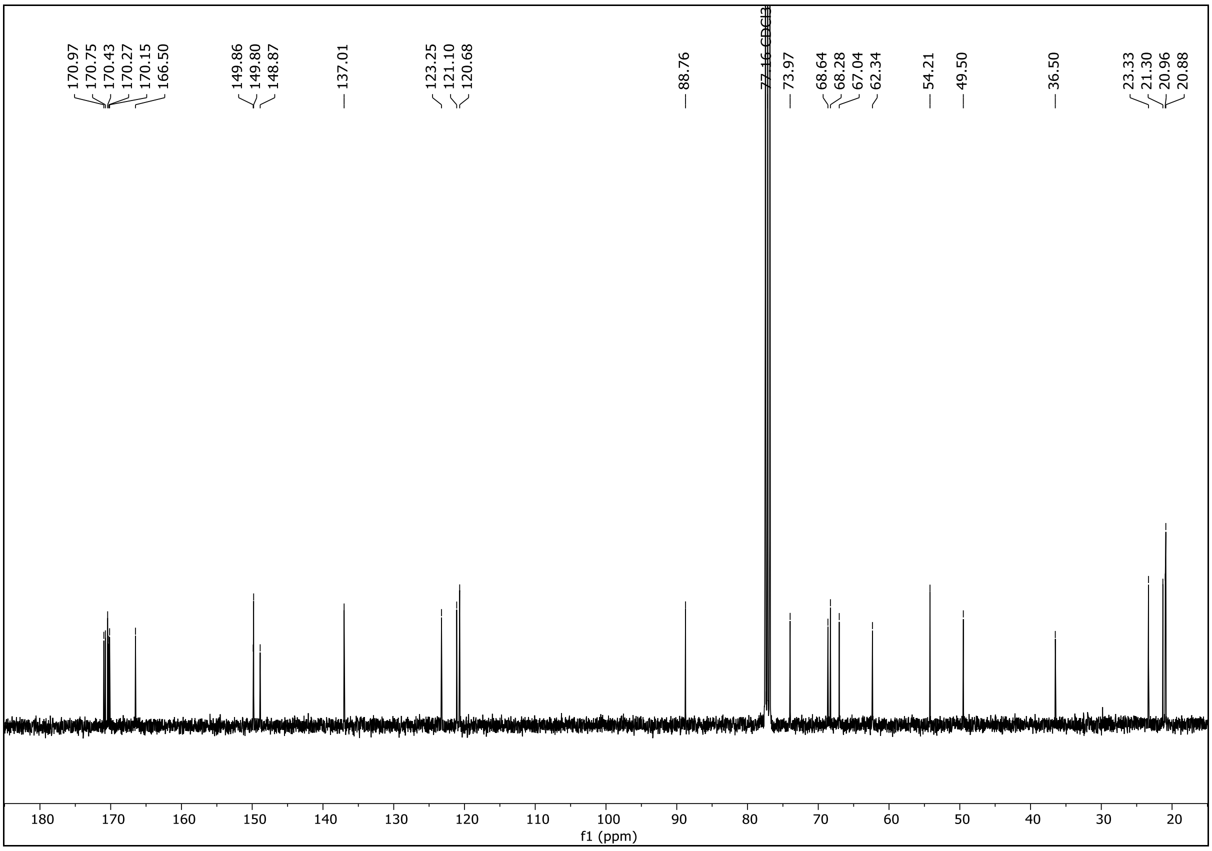

^{1}H NMR (400 MHz, $CDCl_3$) spectrum of compound **2e**

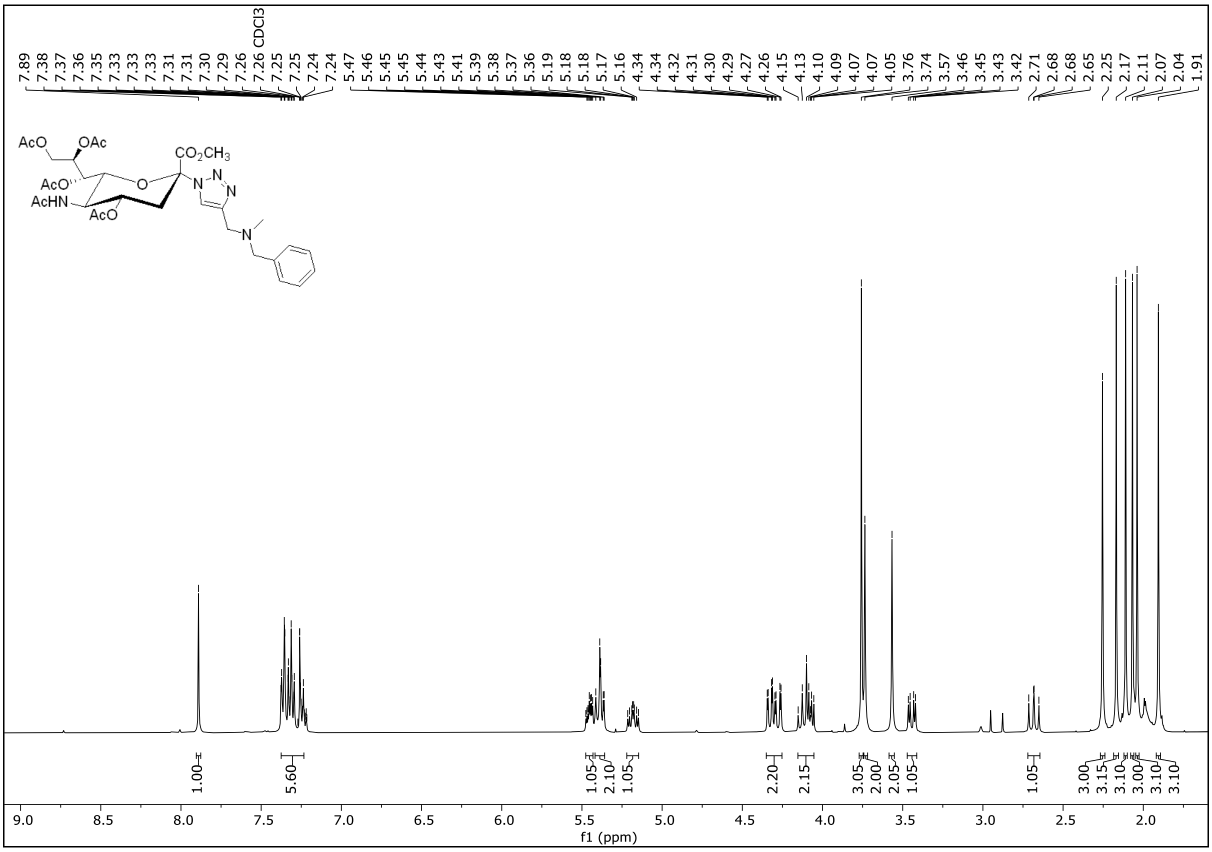

^{13}C NMR (101 MHz, $CDCl_3$) spectrum of compound **2e**

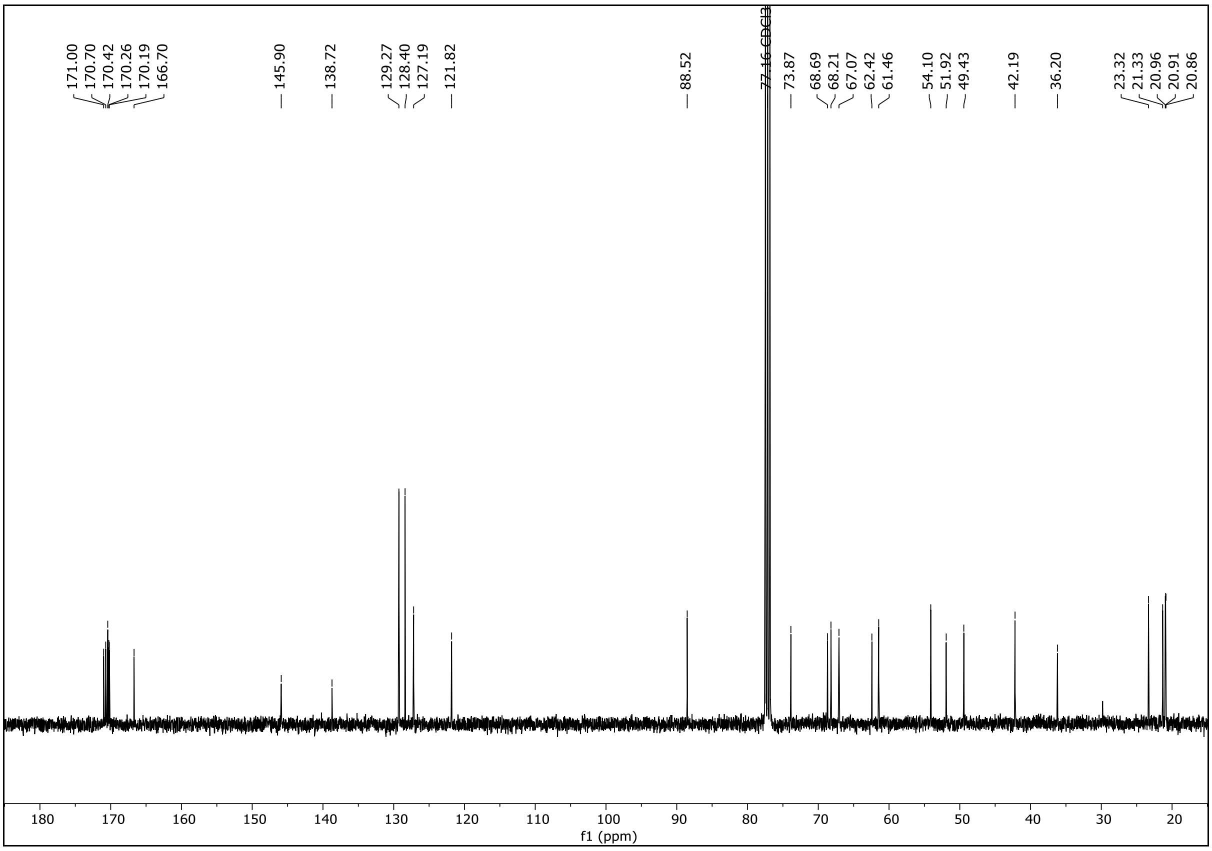

^1H NMR (400 MHz, $CDCl_3$) spectrum of compound **2f**

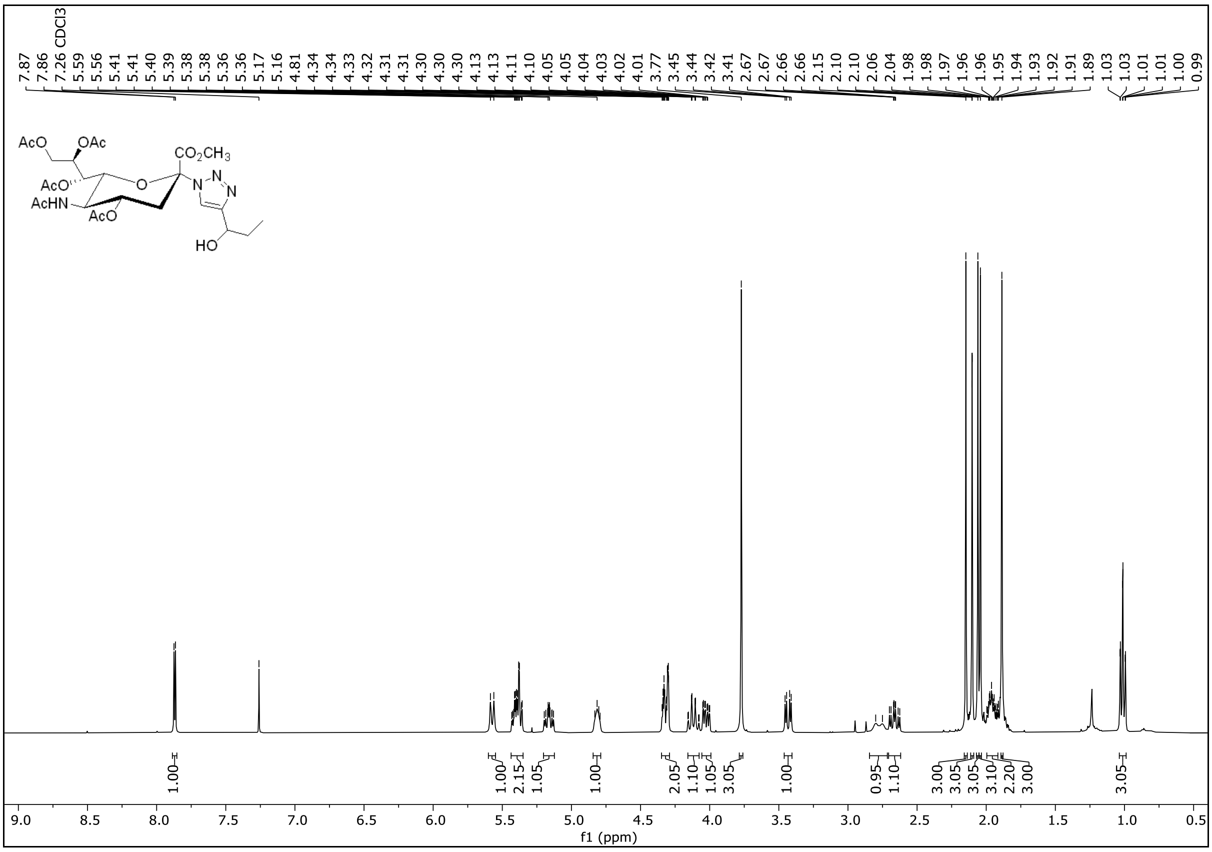

^{13}C NMR (101 MHz, $CDCl_3$) spectrum of compound **2f**

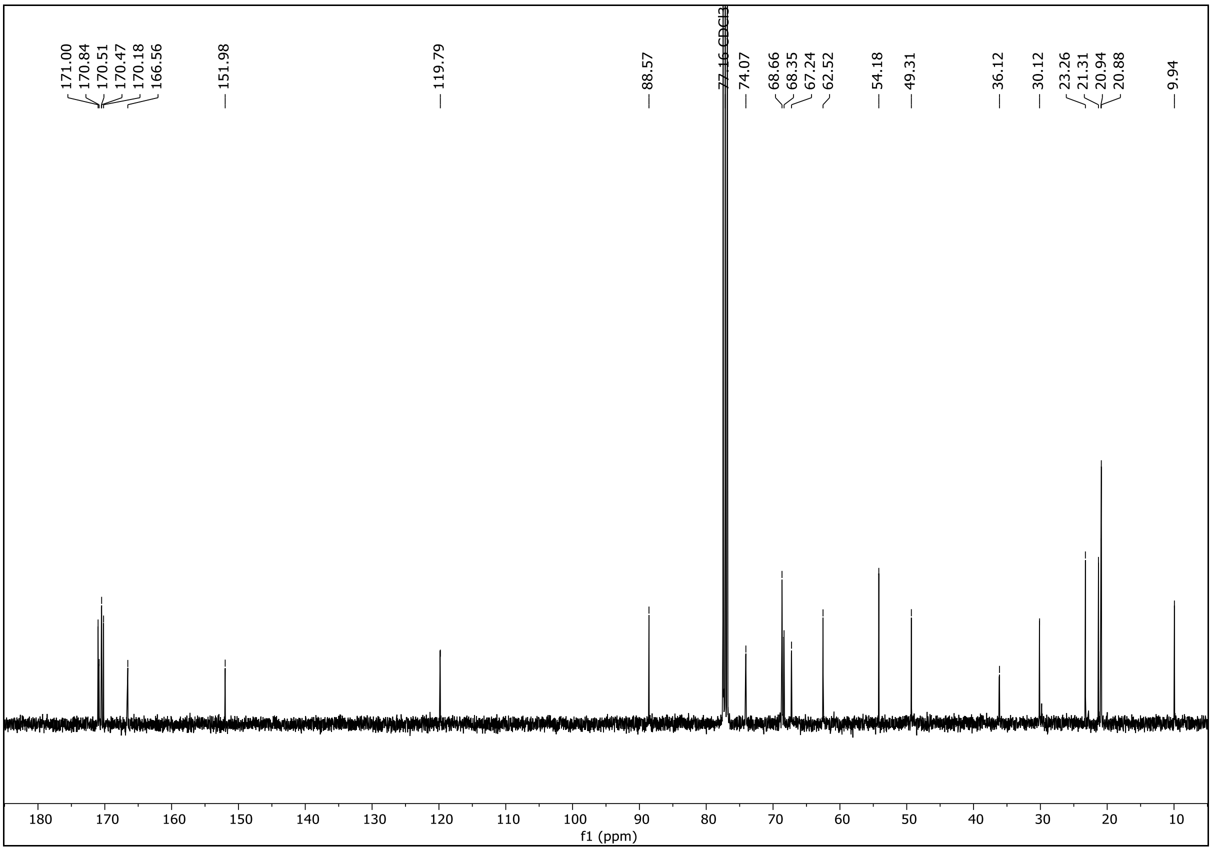

^{1}H NMR (400 MHz, $CDCl_3$) spectrum of compound **2g**

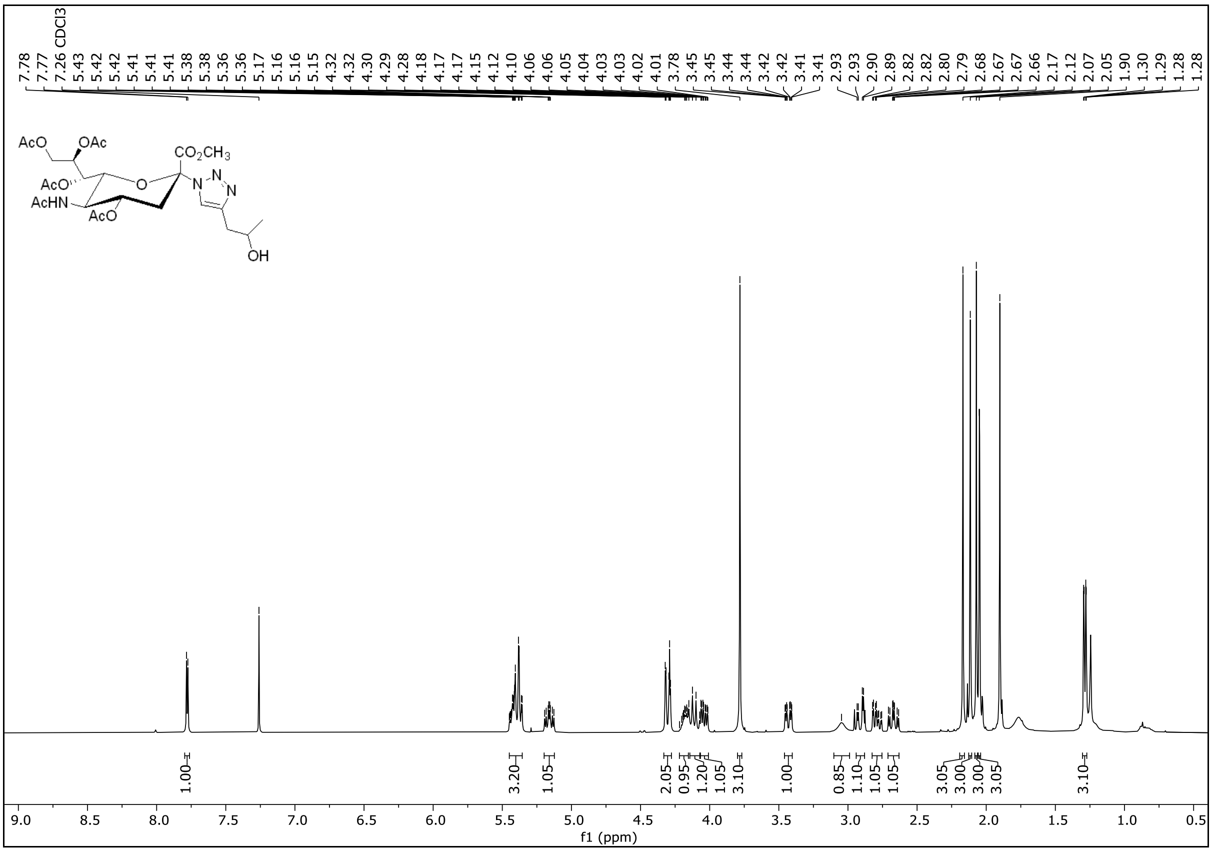

^{13}C NMR (101 MHz, $CDCl_3$) spectrum of compound **2g**

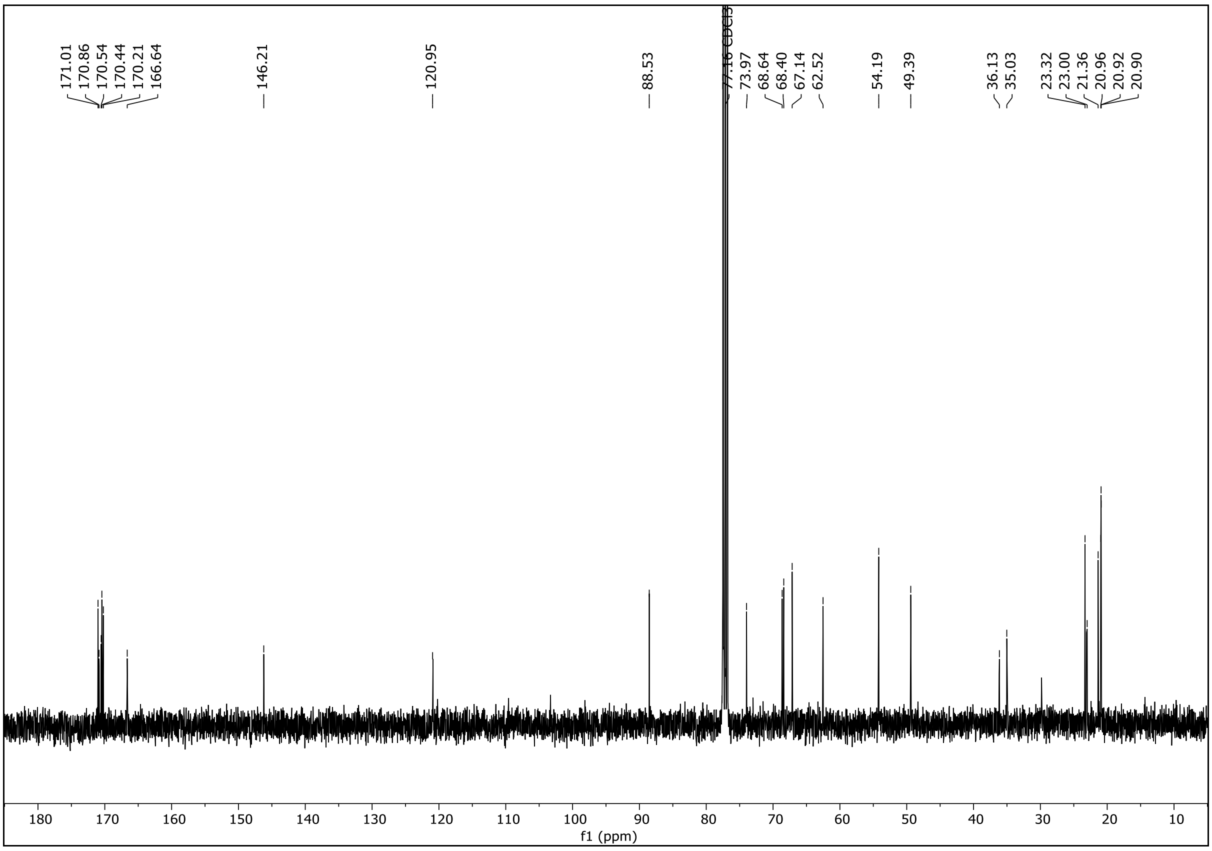

^1H NMR (400 MHz, $CDCl_3$) spectrum of compound **2h**

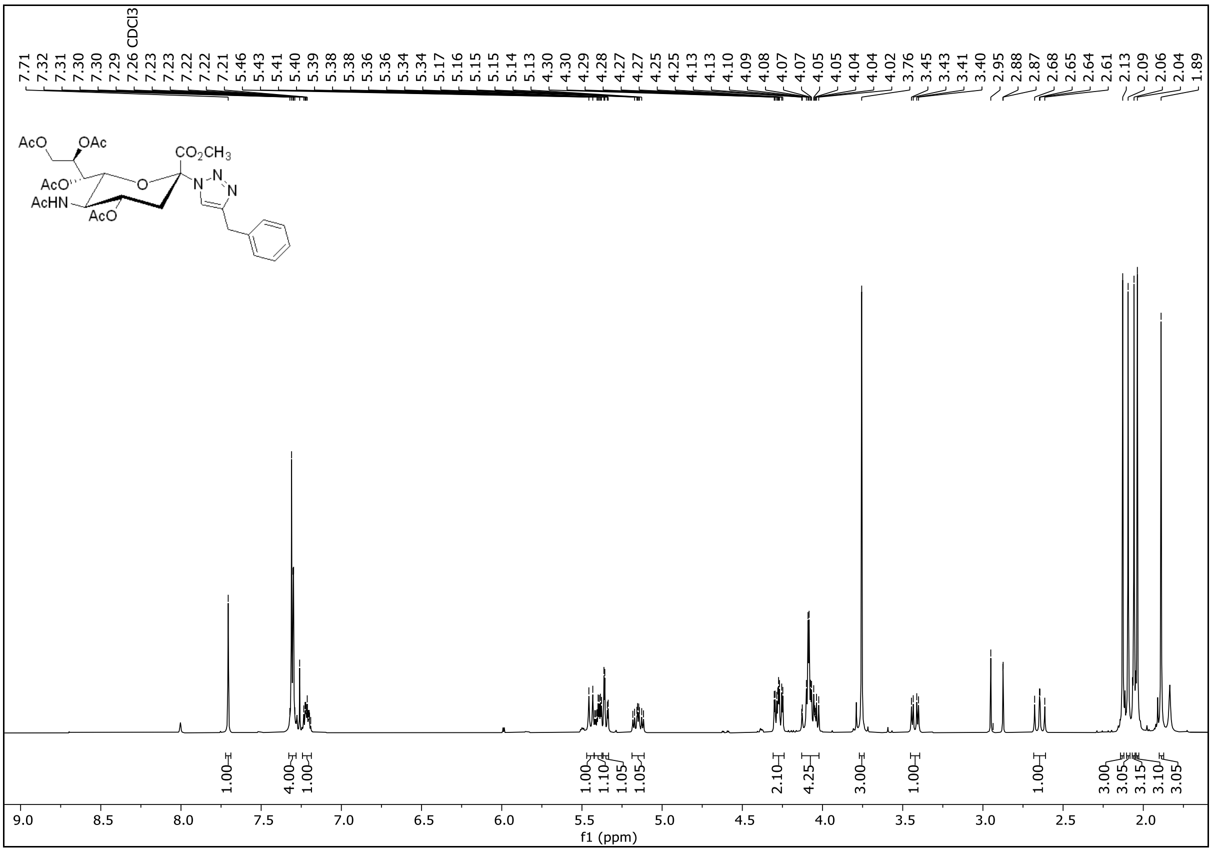

^{13}C NMR (101 MHz, $CDCl_3$) spectrum of compound **2h**

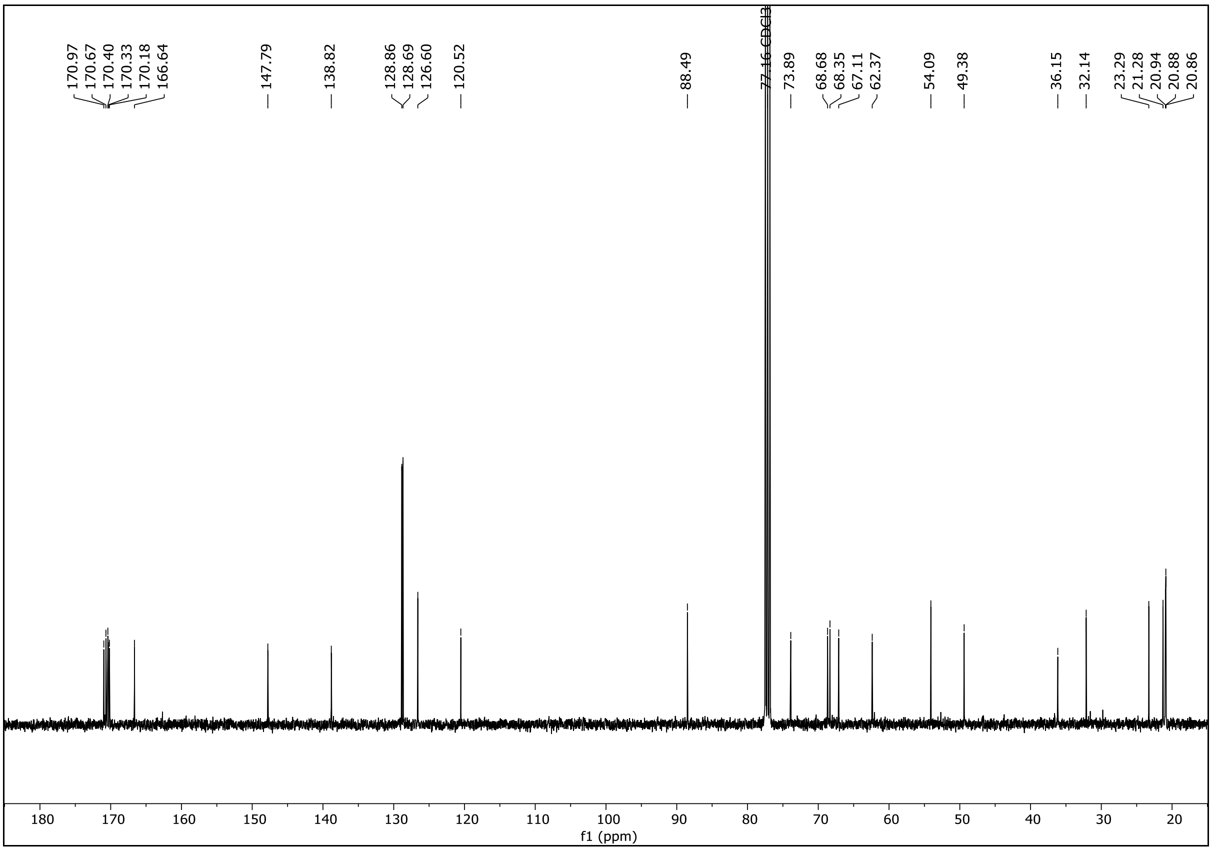

^{1}H NMR (400 MHz, D_2O) spectrum of compound **3a**

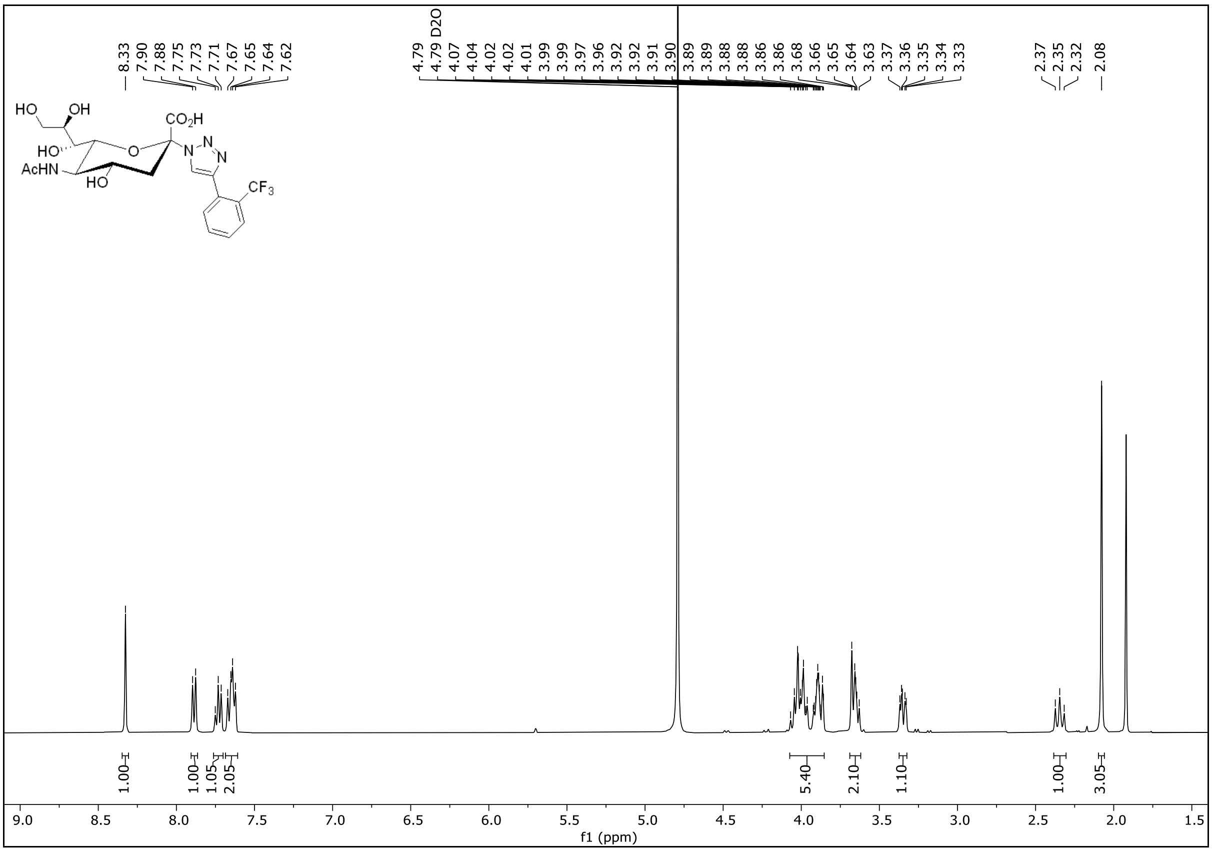

^{13}C NMR (101 MHz, D_2O) spectrum of compound **3a**

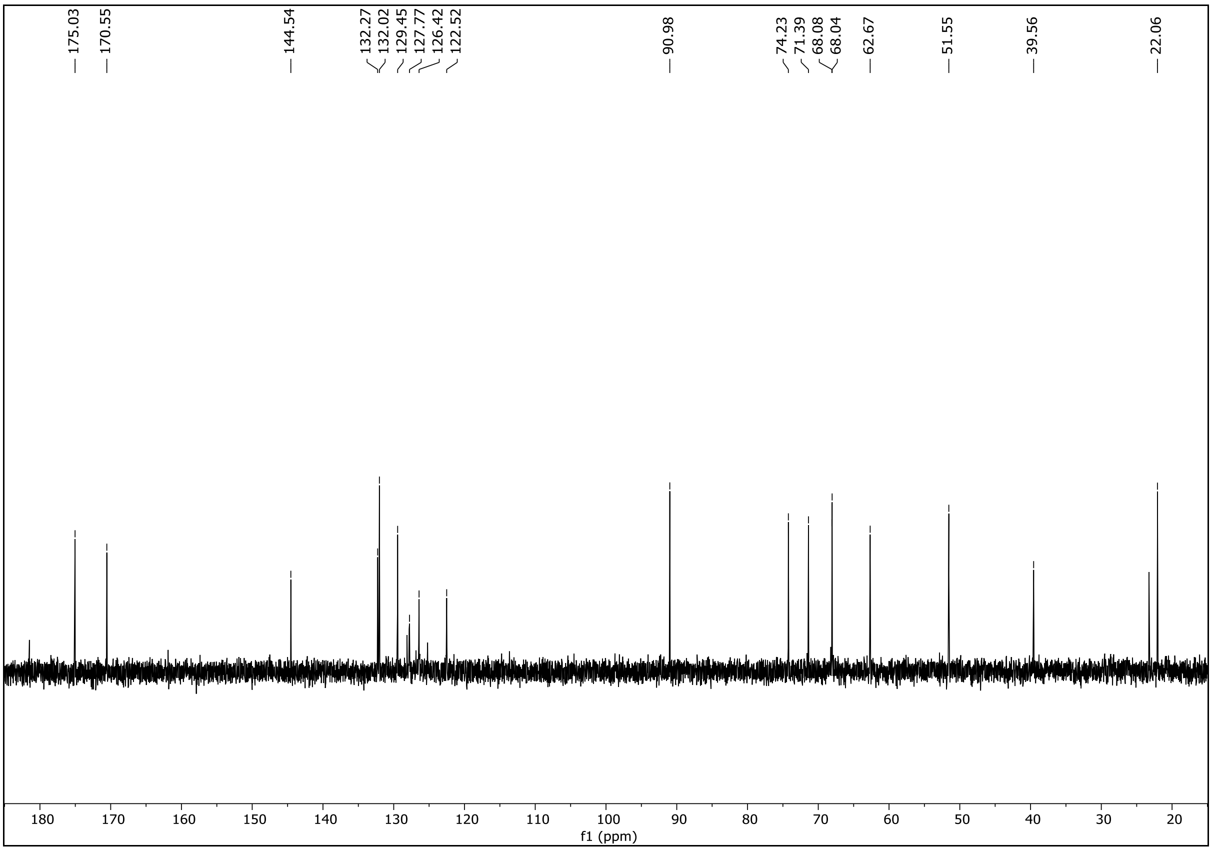

^{1}H NMR (400 MHz, D_2O) spectrum of compound **3b**

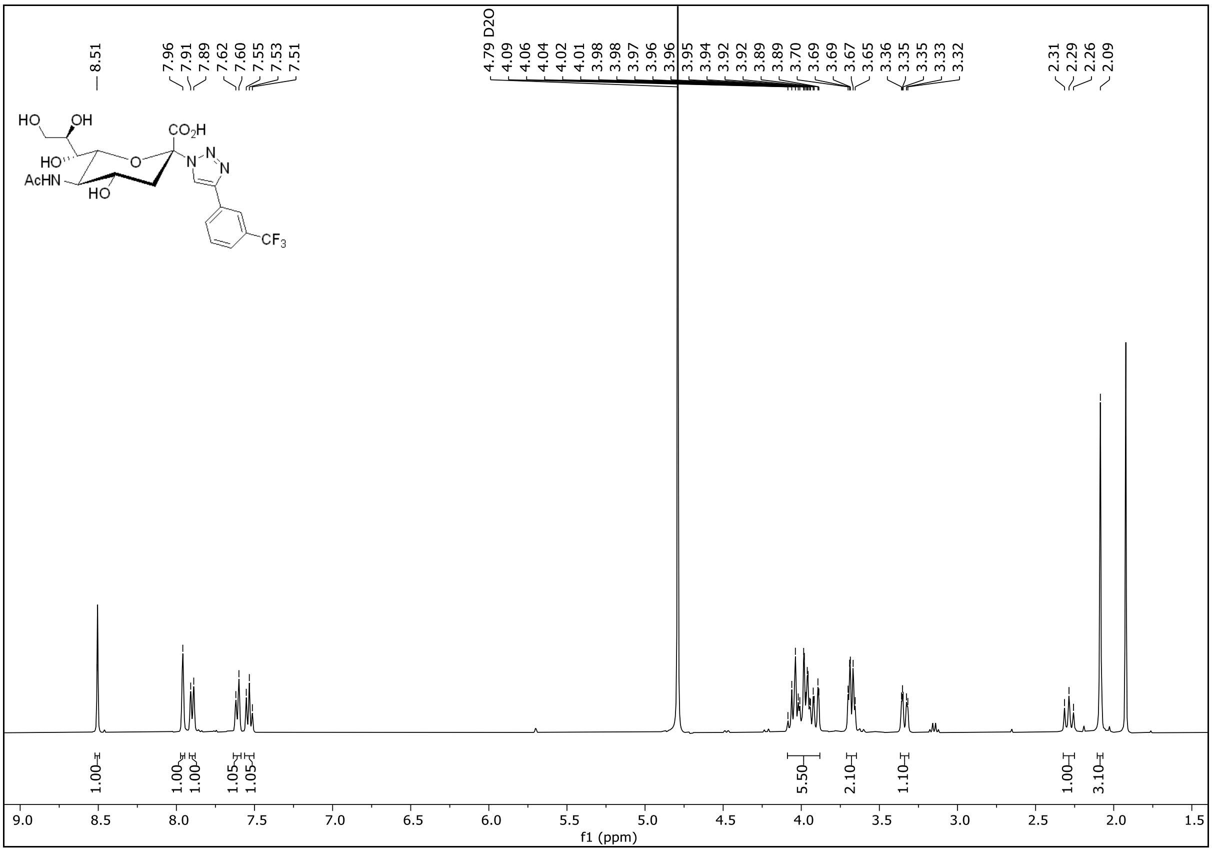

^{13}C NMR (101 MHz, D_2O) spectrum of compound **3b**

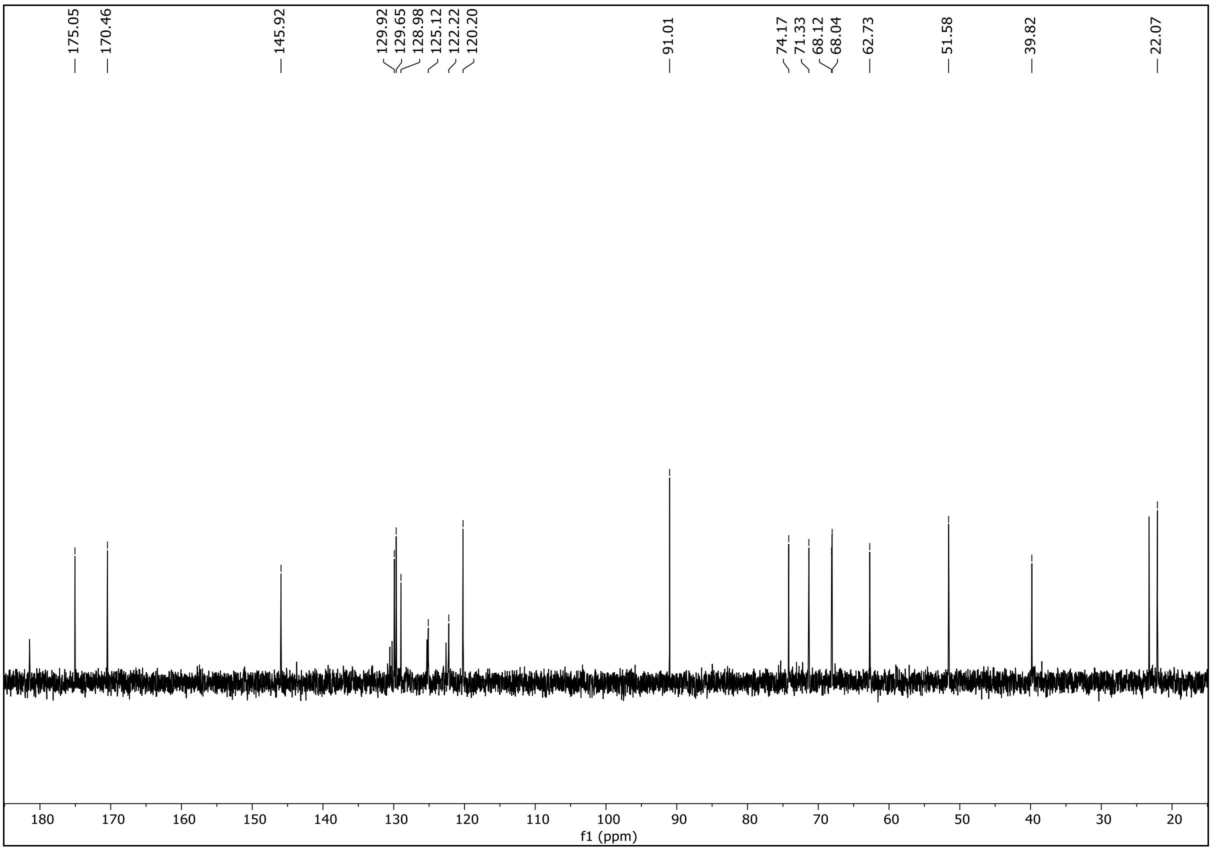

^{1}H NMR (400 MHz, D_2O) spectrum of compound **3c**

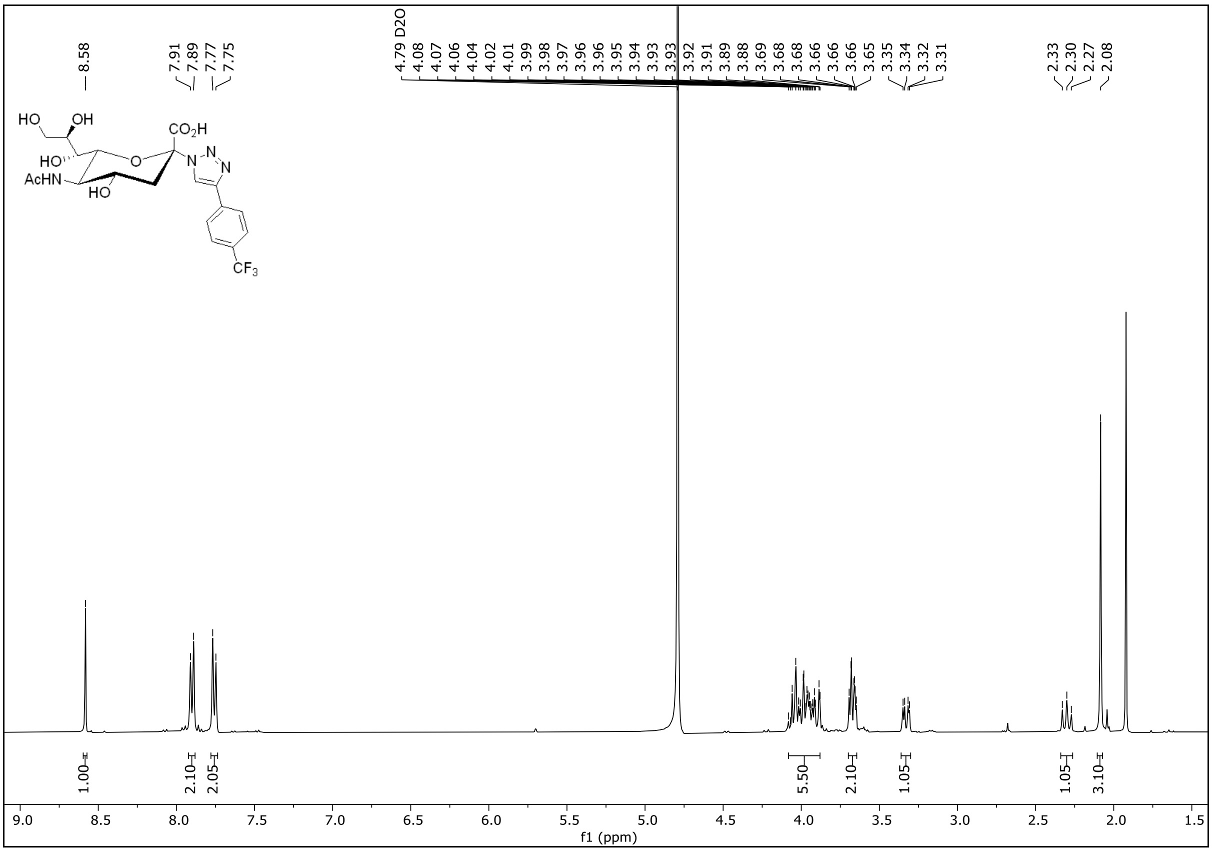

^{13}C NMR (101 MHz, D_2O) spectrum of compound **3c**

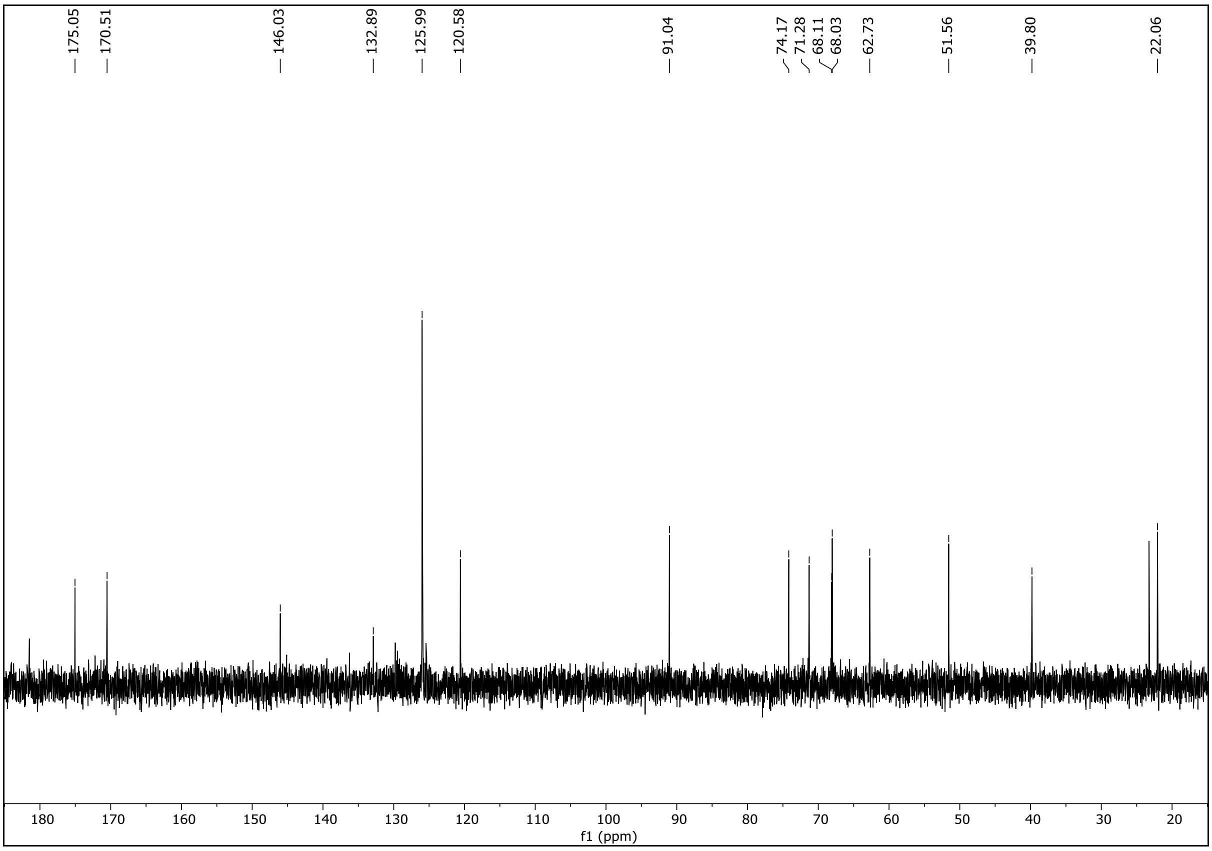

^{1}H NMR (400 MHz, D_2O) spectrum of compound **3d**

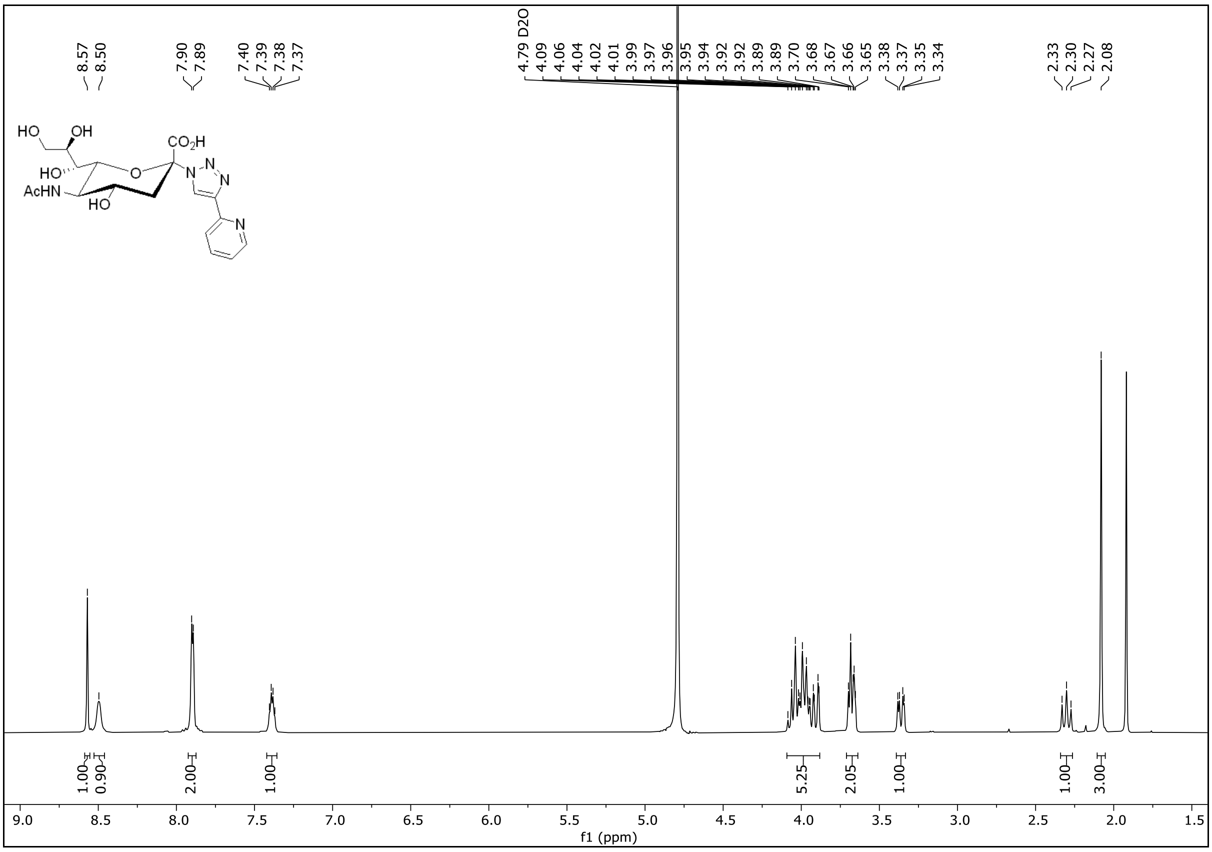

^{13}C NMR (101 MHz, D_2O) spectrum of compound **3d**

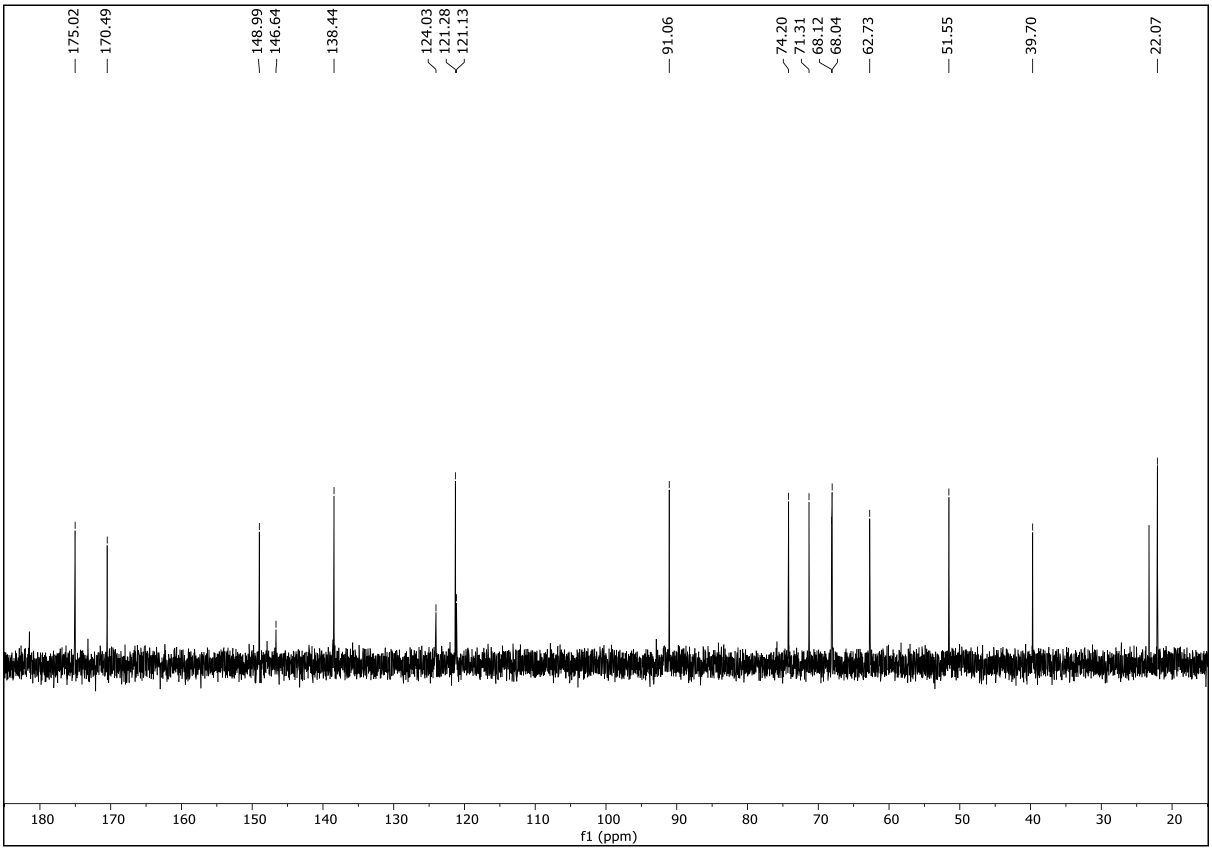

^1H NMR (400 MHz, D_2O) spectrum of compound **3e**

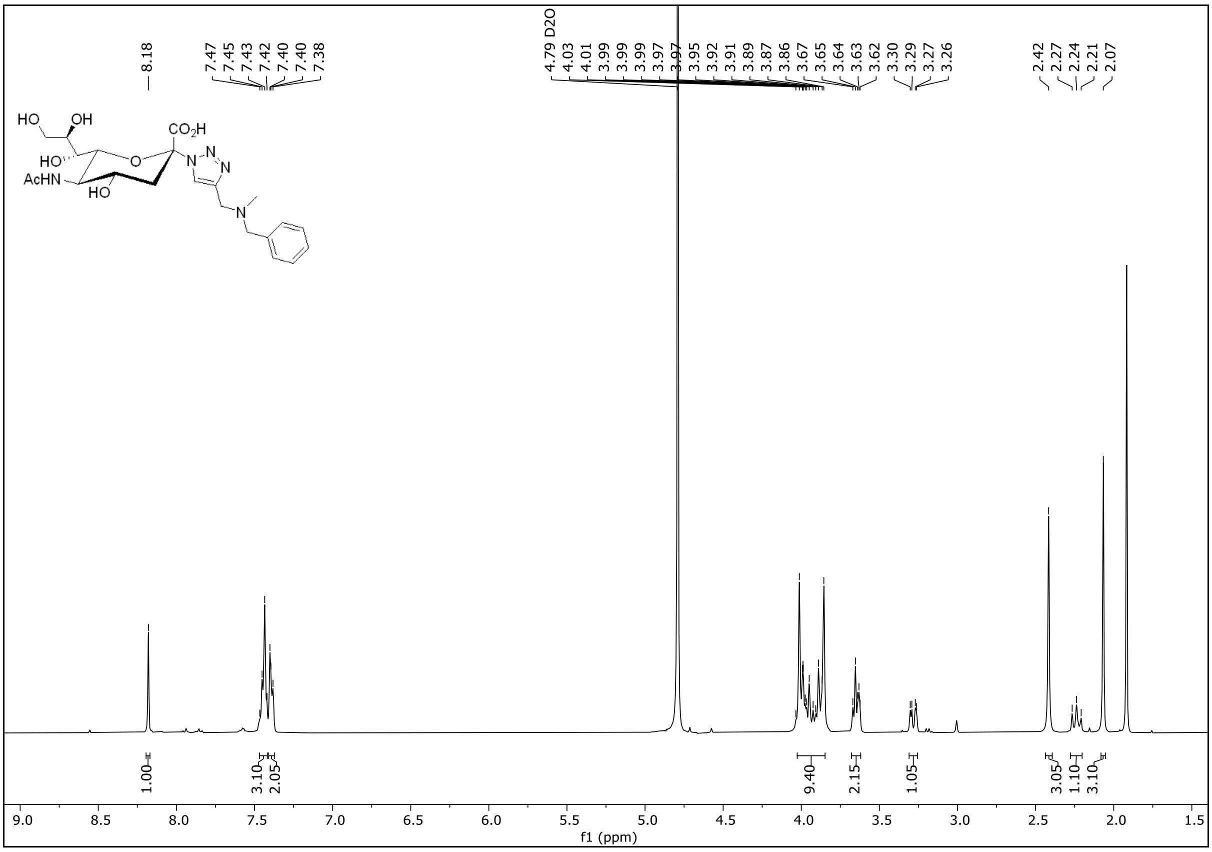

^{13}C NMR (101 MHz, D_2O) spectrum of compound **3e**

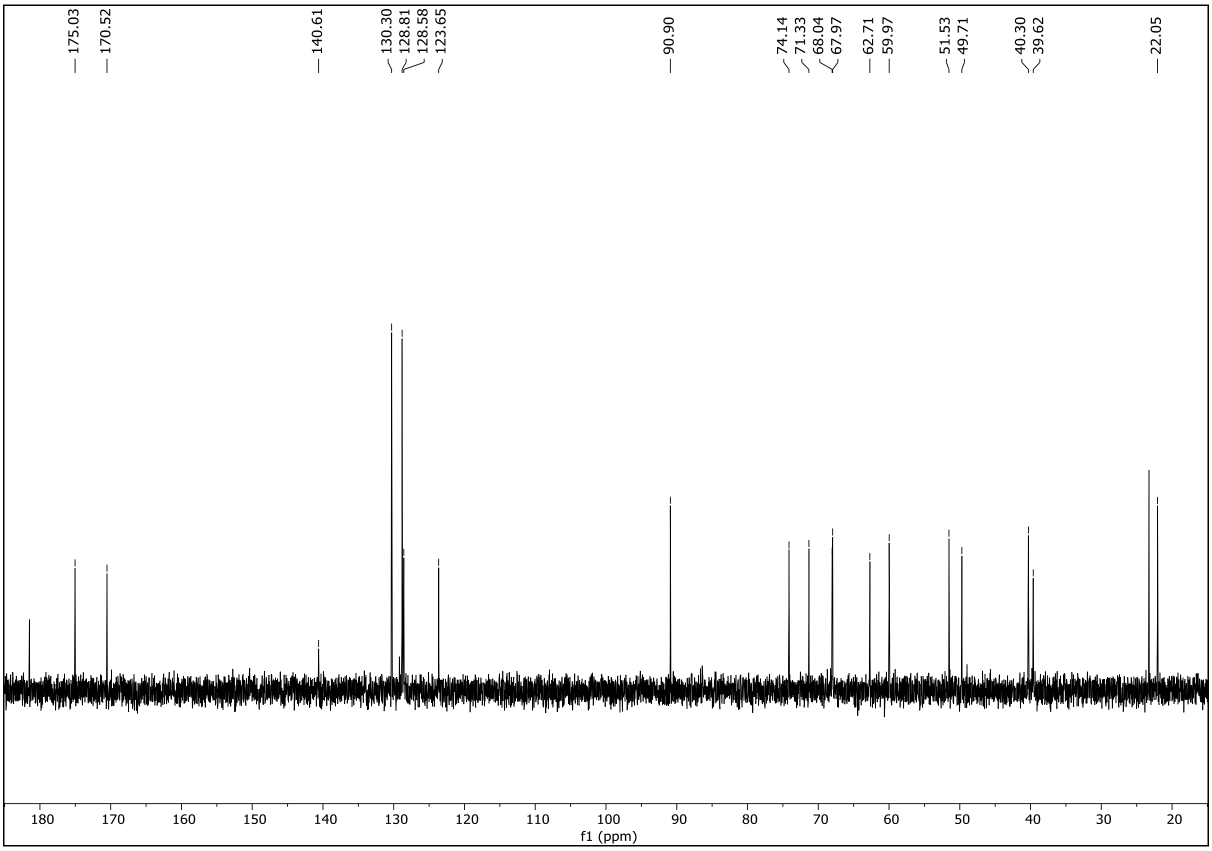

^{1}H NMR (400 MHz, D_2O) spectrum of compound **3f**

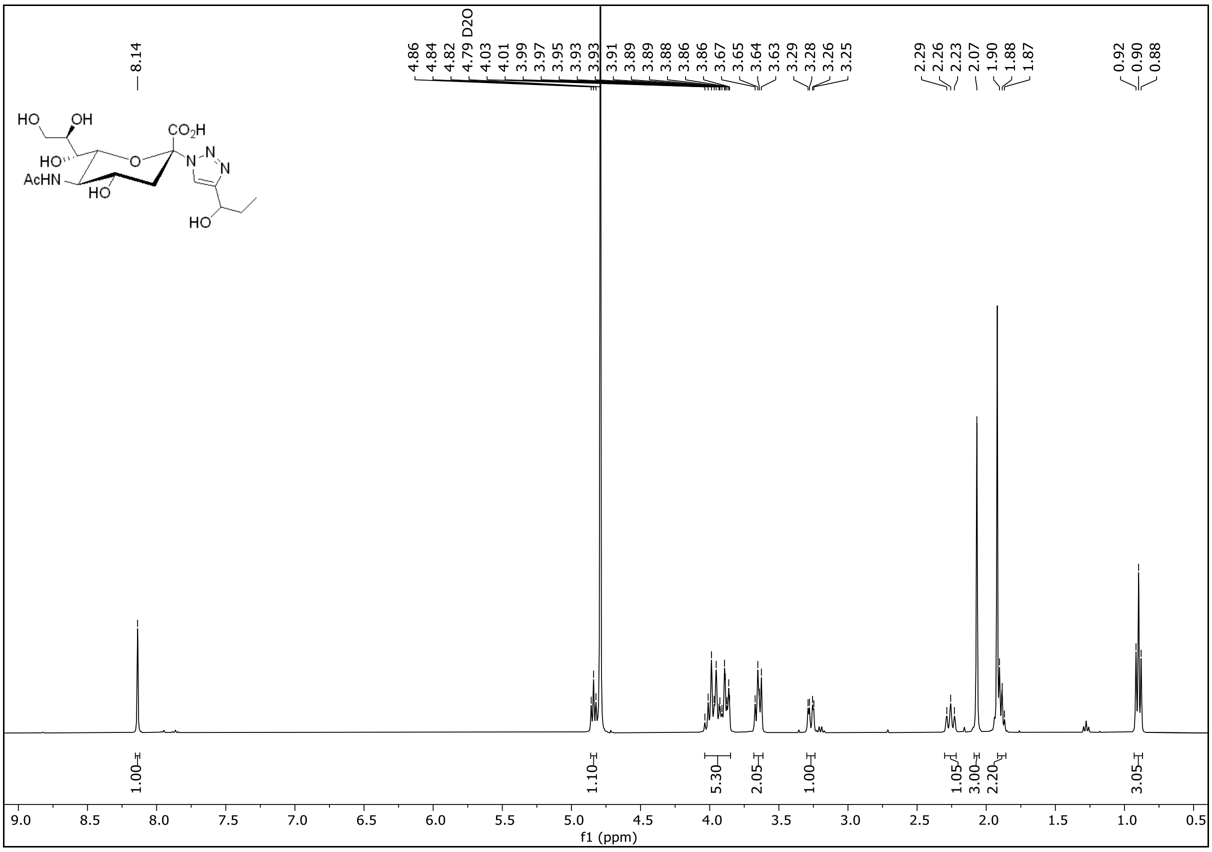

^{13}C NMR (101 MHz, D_2O) spectrum of compound **3f**

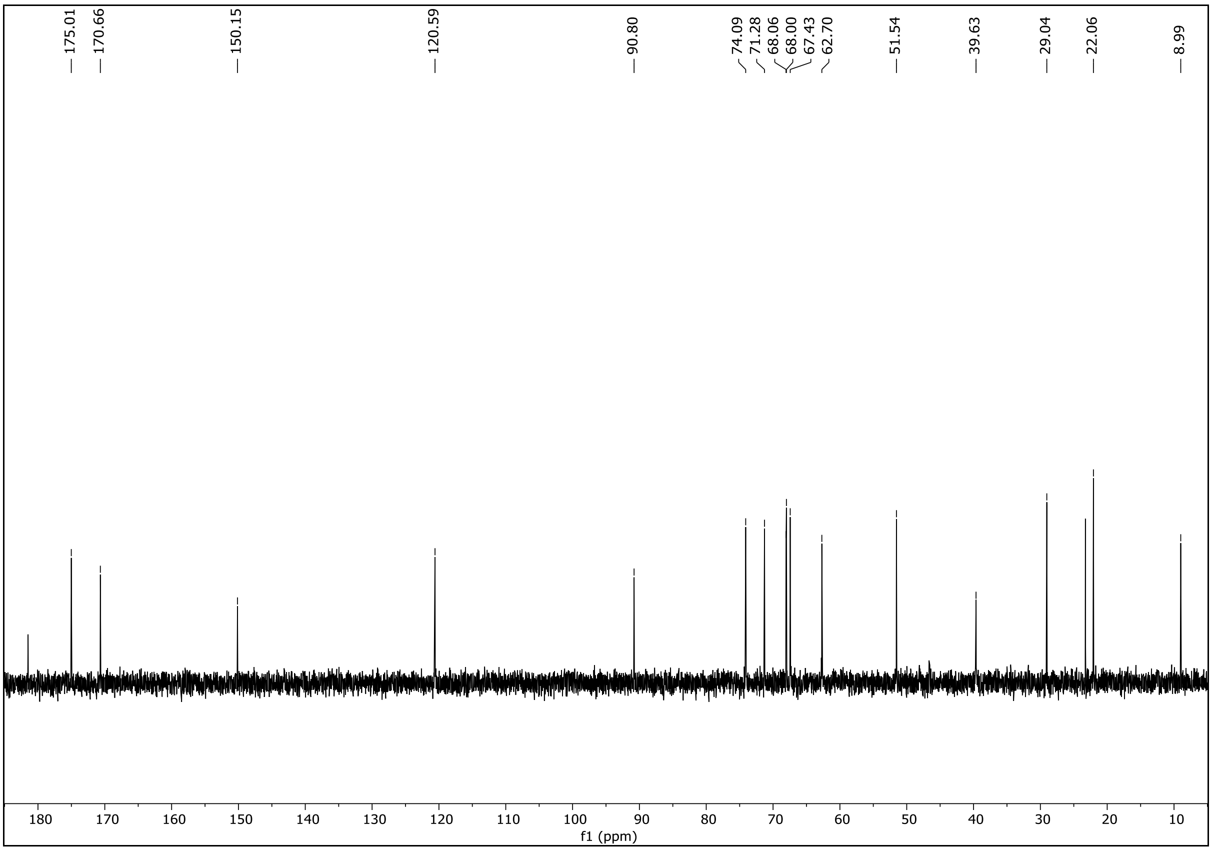

^{1}H NMR (400 MHz, D_2O) spectrum of compound **3g**

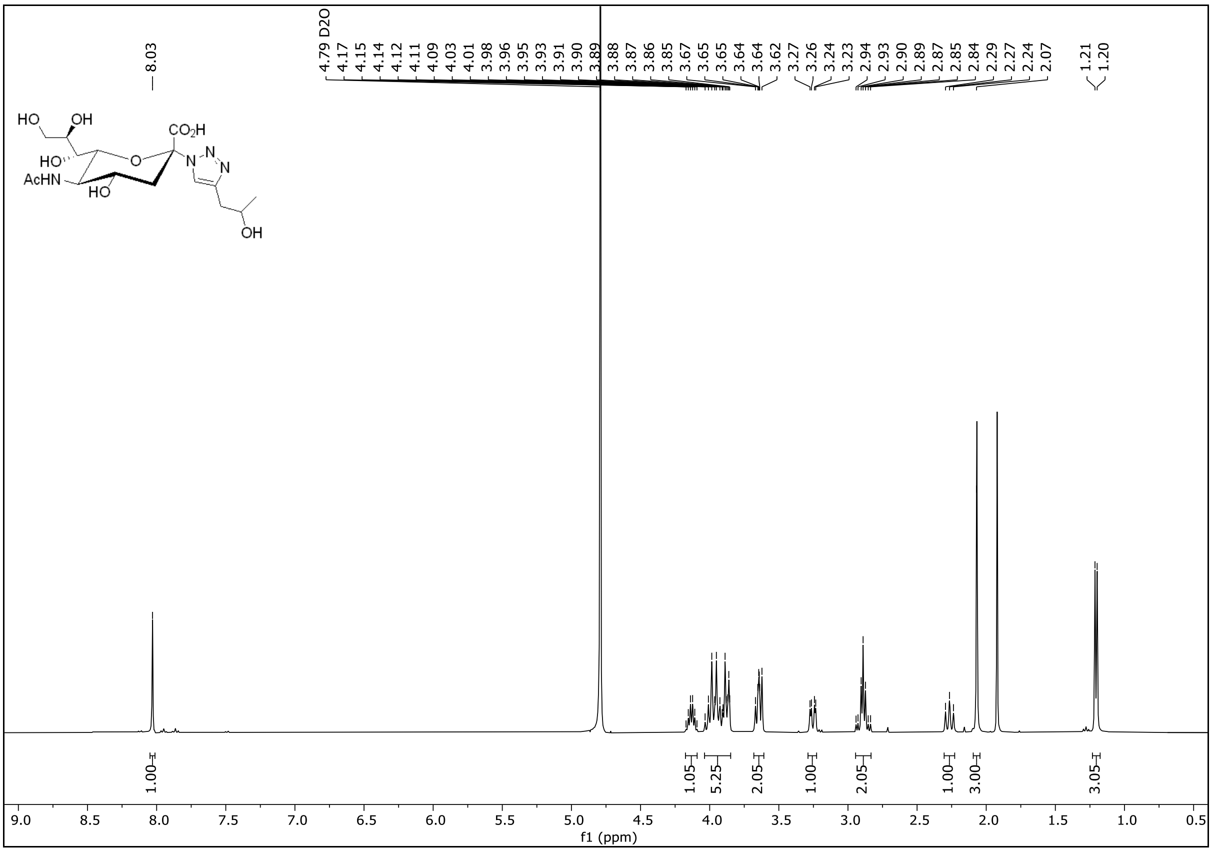

^{13}C NMR (101 MHz, D_2O) spectrum of compound **3g**

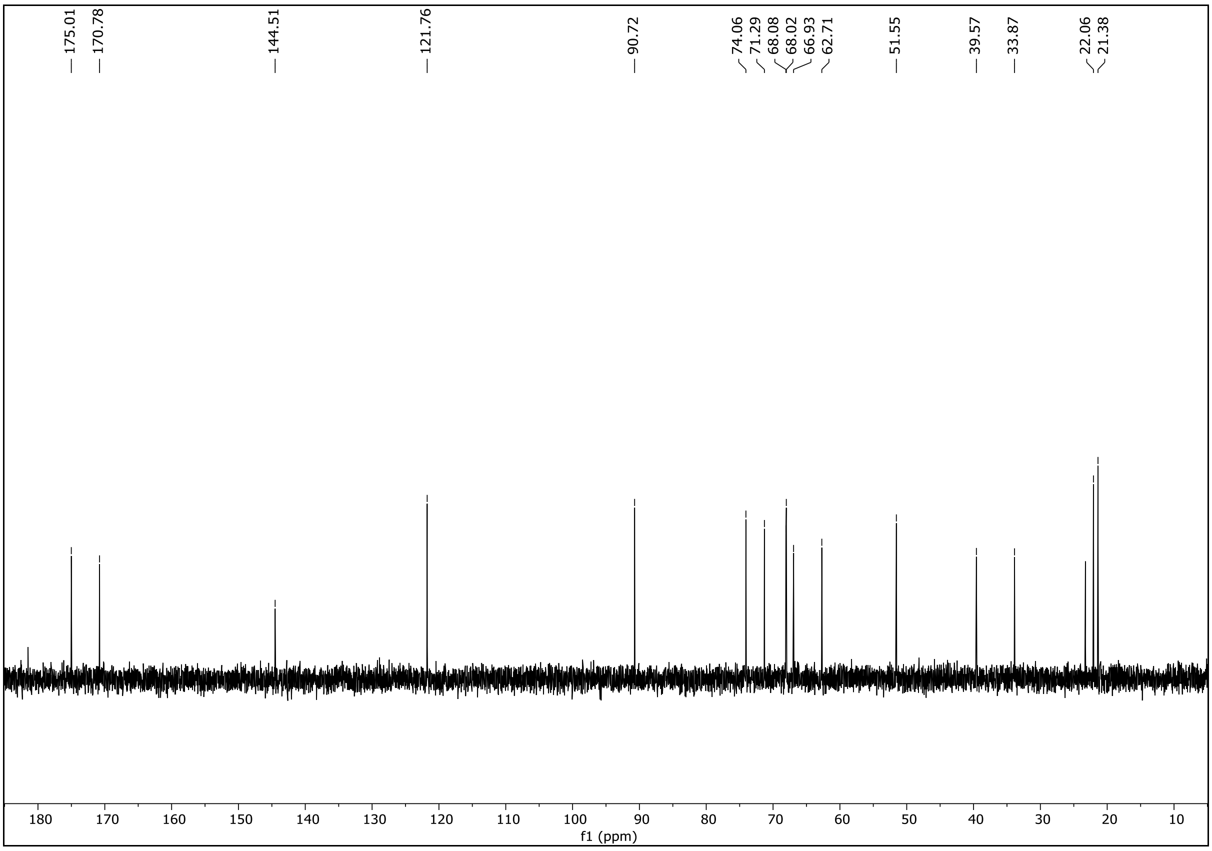

^1H NMR (400 MHz, D_2O) spectrum of compound **3h**

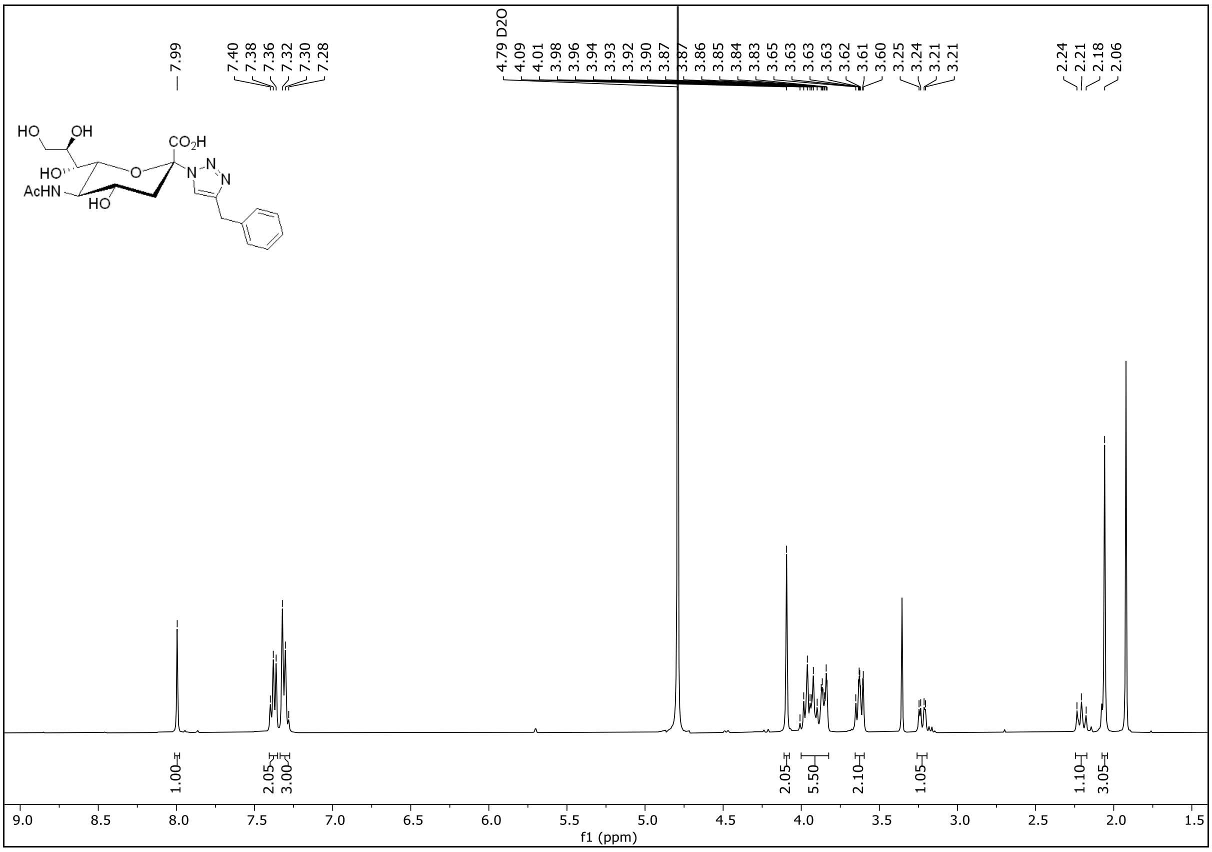

^{13}C NMR (101 MHz, D_2O) spectrum of compound **3h**

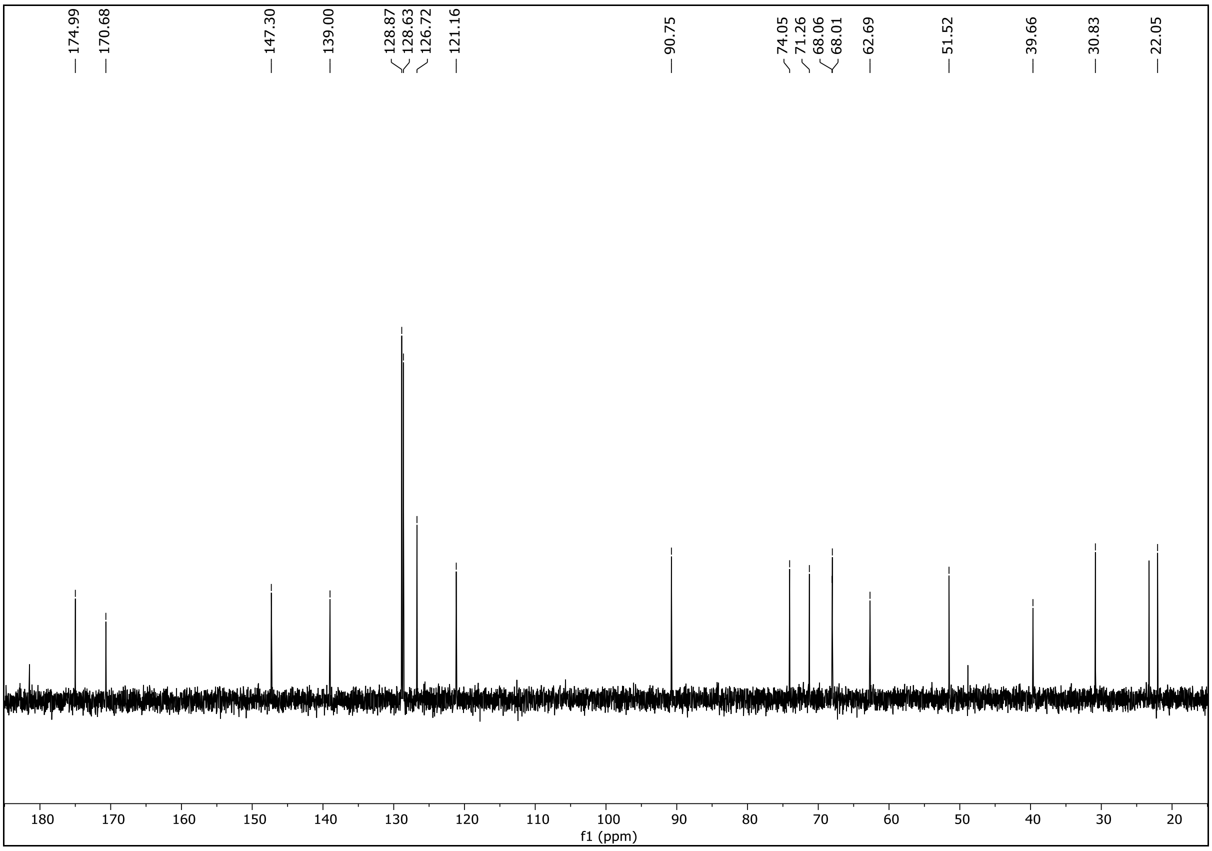

Table S1. Calculated LogP* of compounds **3a–h**.

Compound	Structure	LogP
**3a**	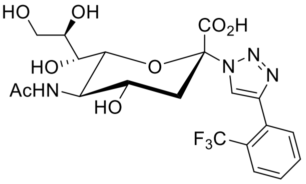	**0.08**
**3b**	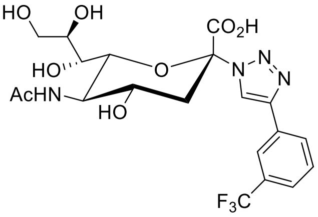	**0.08**
**3c**	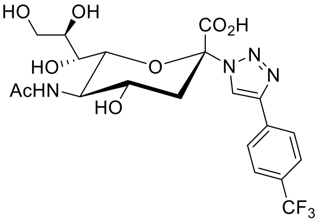	**0.08**
**3d**	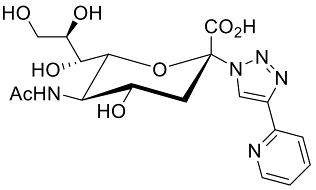	**-1.76**
**3e**	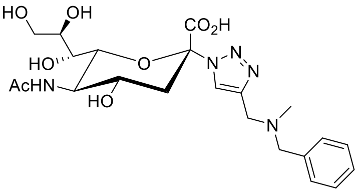	**-0.84**
**3f**	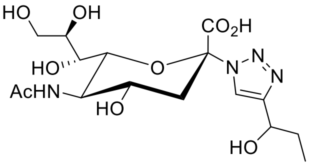	**-2.29**
**3g**	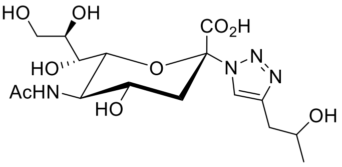	**-2.67**
**3h**	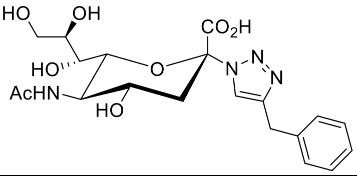	**-0.56**

* Calculated with CambridgeSoft software (ChemDraw's Chemical Properties).
